# Combined effect of Poynting-Robertson (P-R) drag, oblateness and radiation on the triangular points in the elliptic restricted three-body problem

**DOI:** 10.1038/s41598-024-61935-1

**Published:** 2024-05-21

**Authors:** Jagadish Singh, Blessing Samuel Ashagwu

**Affiliations:** 1 Department of Mathematics, Ahmadu Bello University, Zaria, Nigeria; 2 Department of Mathematics, Air Force Institute of Technology (AFIT), Kaduna, Nigeria

**Keywords:** Celestial mechanics, Elliptic restricted three-body problem, Oblateness, Radiation, Poynting-Robertson (P-R) drag, Astronomy and planetary science, Mathematics and computing

## Abstract

This study investigates the motion of a test particle around triangular equilibrium points in the elliptic restricted three-body problem (ER3BP) under the influence of the two oblate and radiating primaries having Poynting-Robertson (P-R) drag. It is observed that the position of triangular points of the problem is affected by oblateness, radiation pressure, eccentricity, semi-major axis and Poynting-Robertson (P-R) drag. The stability of these points is demonstrated analytically by the Routh-Hurwitz criterion. It is seen that they are unstable under the combined effect of involved parameters. The effect of these parameters on the position of triangular points is examined numerically using the binary systems, 61 Cygni and Archird. The results obtained by these binary systems can be used to broaden the scope of interest in astronomy, astrophysics, space science and celestial mechanics in general.

## Introduction

The elliptic restricted three-body problem (ER3BP) describes the motion of infinitesimal mass (test particle) under the influence of two massive bodies (primaries) moving under their mutual gravitational attraction in elliptic orbits. The photogravitational elliptic restricted three-body problem with Poynting-Robertson (P-R) drag and oblateness refers to a complex dynamical system that combines the effects of gravitational forces, radiation pressure, Poynting-Robertson (P-R) drag, and the oblateness of one or both of the primary bodies. The elliptic restricted three-body problem consists of five equilibrium points $L_{i}$, ($i = 1, \ldots ,5$), the first three of which are said to be collinear; and the remaining two are known as triangular points. Studies on elliptic-restricted three-body problems have been carried out in various areas^1–17^.

Several perturbations have been considered in the study of the circular restricted three-body problem (CR3PB), viz oblateness, radiation, triaxiality, radiation, Poynting Robertson (P-R drag) etc. These perturbations can cause a change in the stability of the system. This has motivated several researchers such as; ^8,11,18–31^. On these aforesaid foundations, a need arises to develop a model that defines the elliptic restricted three-body problem (ER3BP) with an infinitesimal mass under the influence of oblate and radiating primary bodies having Poynting-Robertson (P-R) drag.

Radzievskii^32^ was the first to study the restricted three-body with the radiation pressure force and found the out-of-plane equilibrium points, L_6_ and L_7_. Later, Chernikov^33^ introduced the Poynting-Robertson (P-R drag) drag.

The Poynting–Robertson drags effect, also called P–R drag, was named after John H. Poynting and Howard P. Robertson. Robertson^34^ stated the effect as a process by which solar radiation causes a dust grain orbiting a star to lose angular momentum relative to its orbit around the star, while Poynting^35^ on the other hand described the effect based on the luminiferous theory. Given the importance of the problem, several authors like ^36–39^ have carried out their studies taking Poynting–Robertson (P-R) drag into consideration. Recently, Vincent and Kalantonis^36^ studied the motion around the equilibrium points in the photogravitational restricted three-body problem under the effects of Poynting–Robertson drags, circumbinary belt and triaxial primaries with an oblate infinitesimal body: Application on Archird binary system. In the study of a circular restricted three-body problem, Singh and Emmanuel^37^ examined the behaviour of the equilibrium points in the vicinity of a smaller triaxial body together with a radiating companion having Poynting–Robertson (P-R) drag while Singh and Amuda^38^ investigated the stability of equilibrium points in the circular restricted three-body problem when the bigger primary is an oblate spheroid and a smaller one is a radiating primary having Poynting–Robertson drag. Singh and Amuda^39^ examined the effect of Poynting–Robertson (P-R) drag, radiation, and oblateness on motion around the triangular equilibrium points in the photogravitational restricted three-body problem. It was observed that the triangular points are unstable due to the presence of at least one characteristic complex root with the positive real part.

In this present study, we have extended the work of Singh and Amuda ^39^ to carry out an analytic and numerical study on the motion of the test particle (infinitesimal mass) to ascertain the influence of the oblateness and radiation pressure on the locations and stability of the equilibrium points in the elliptic restricted three-body problem having Poynting-Robertson drag for the binary system: 61 Cygni and Archird. The analytic study is carried out using the Routh-Hurwitz criterion for stability which states that; for a system to be stable, all the roots of the first column must have the same sign and if it does not have the same sign or there is a sign change, then the number of sign changes in the first column is equal to the number of roots with positive real parts (i.e. all minor matrices must be greater than zero). The numerical compilations of this work are done with the help of MATHEMATICA 12.1 software for the systems: 61 Cygni and Archird. They are both binary stars. 61 Cygni is a binary star system consisting of a pair of K-type dwarf stars that orbit each other about 659 years of apparent magnitude 5.20 and 6.05 respectively. They can be seen with binoculars in city skies or with the naked eye in rural areas without light pollution (Strand). Archird also called Eta Cassiopeiae, is a binary star system located in the northern circumpolar. It is situated at around 19.42 light-years away from the Sun. The Archird star system is composed of the primary star Eta Cassiopeiae A, and its companion Eta Cassiopeiae B. T. Archird is one of the fainter stars in the constellation. Archird was formed around 5.4 ± 0.9 billion years ago. What makes Archird special is that it is similar to the Sun and it is among the closest stars to us.

In a recent paper, Elipe^40^ has stated that the assumption of the primaries being oblate spheroids is only applicable to the circular restricted three-body problem (CR3BP). In the context of the present paper, it is worth to note that the elliptic restricted three-body problem (ER3BP) in which the eccentric anomaly is considered as an independent variable (instead of the true anomaly), can be dynamically treated as the circular restricted three-body problem (CR3BP). (see Singh and Umar^8^).

The arrangement of this paper is in seven parts: part 1 is the introduction; part 2 presents the equations of motion of the problem; part 3 demonstrates the positions of triangular points; part 4 analyzes their linear stability, and in part 5, the problem is explored numerically. In parts 6 and 7, the discussion and conclusion are provided respectively.

## Derivation of equations of motion

**Figure 1:**
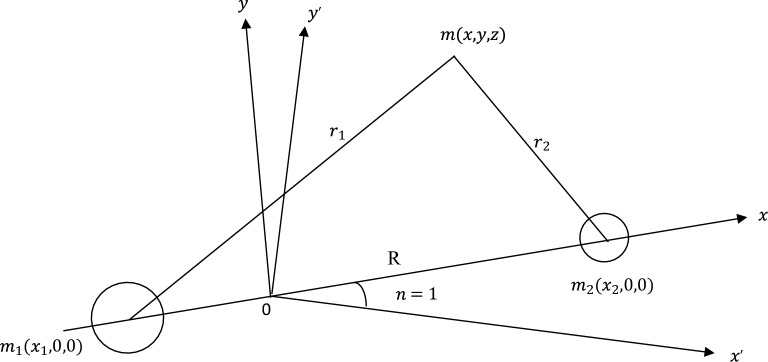
Bigger primary $m_{1}$ and smaller primary $m_{2}$ moving in an elliptic orbits about their common centre of mass unperturbed by the infinitesimal mass m.

Consider a rotating frame of reference $$0xyz$$ where $$0$$ is the origin at the center of mass of the two primaries m_1_ and m_2_ (Szebehely^41^). The $$x - axis$$ lies along the line joining the two finite masses $$m_{1} {\text{and }}m_{2}$$. The $$y - axis$$ lies in the plane of the orbits of the finite bodies and is perpendicular to the $$x - axis$$. The $$z - axis$$ is perpendicular to the orbital plane of the finite bodies. Let $$r_{1}$$ and $$r_{2}$$ be the respective distances of $$m_{1} and m_{2} $$ of the infinitesimal body $$m$$ from the bigger and smaller primaries positioned at $$\left( {x_{1} , 0, 0} \right)$$ and $$\left( {x_{2} , 0, 0} \right)$$ respectively as shown in Fig. 1. Then, the equations of motion of the tiny mass in the sidereal coordinate according to Singh and Umar^8^, can be given as;

1 $$ \ddot{x} = \frac{\partial U}{{\partial x}}, \ddot{y} = \frac{\partial U}{{\partial y}}, \ddot{z} = \frac{\partial U}{{\partial z}} $$

2 $$ U = k^{2} \left( {\frac{{m_{1} }}{{r_{1} }} + \frac{{m_{2} }}{{r_{2} }}} \right) $$

$$ R_{i}^{2} = \left( {x - x_{i} } \right)^{2} + \left( {y - y_{i} } \right)^{2} + \left( {z - z_{i} } \right)^{2} , \left( {i = 1,2} \right) $$

where $k^{2}$ is the gravitational constant, we now rotate the rectangular frame of reference about the $z - axis$ so that the primaries always lie on the $x - axis$. The new coordinates are related to the old ones by transformation formulae.

$$ x = X\cos \upsilon - Y\sin \upsilon , y = X\sin \upsilon + Y\cos \upsilon , z = Z $$

where $\upsilon$ is the true anomaly. Then, the equations of motion are given in Eq. (1) in the synodic reference system are given by

3 $$ \begin{gathered} \ddot{X} - 2\dot{Y}\dot{\upsilon } - \dot{\upsilon }^{2} X - \ddot{\upsilon }Y = \frac{\partial U}{{\partial X}} \hfill \\ \ddot{Y} + 2\dot{X}\dot{\upsilon } - \dot{\upsilon }^{2} Y + \ddot{\upsilon }X = \frac{\partial U}{{\partial Y}} \hfill \\ \ddot{Z} = \frac{\partial U}{{\partial Z}} \hfill \\ \end{gathered} $$

Considering the force function $U$ in Eq. (2) we have that

$$ R_{i}^{2} = \left( {X - X_{i} } \right)^{2} + Y^{2} + Z^{2} , \left( {i = 1,2} \right) $$

4 $$ X_{1} = \frac{{ - m_{2} R}}{{m_{1} + m_{2} }}, X_{2} = \frac{{m_{1} R}}{{m_{1} + m_{2} }} $$

where $R$ is the distance between the primaries.

We change the equations of motion from the true anomaly to the eccentric anomaly, we use the following relation by Singh and Umar^8^;

5 $$ \cos \upsilon = \frac{\cos E - e}{{1 - e\cos E}} $$

where $e, E, and \upsilon$ are respectively the eccentricity, eccentric anomaly and the true anomaly of the orbit of the primaries. Then from Eq. (5) we have

6 $$ \sin \upsilon = \frac{{\left( {1 - e^{2} } \right)^{\frac{1}{2}} \sin E}}{1 - e\cos E} $$

Differentiating Eq. (5) for time $t$ we obtain

7 $$ - \sin \upsilon .\dot{\upsilon } = \frac{{\left( {e^{2} - 1} \right)\sin E.\dot{E}}}{{\left( {1 - e\cos E} \right)^{2} }} $$

By Kepler’s equation, we obtain

8 $$ n\left( {t - T} \right) = E - e\sin E $$

where $n$ and $T$ are, the mean motion and the period of the elliptic orbit respectively. Then, the distances between the primaries are given as;

9 $$ R = a\left( {1 - e\cos E} \right) $$

where ‘$a$’ is the semi-major axis of the elliptic orbit of $m_{2}$ around $m_{2}$. Differentiating Eq. (8) With $t$ and considering Eq. (9) we obtain

10 $$ \dot{E} = \frac{na}{R}, \ddot{E} = \frac{{ - en^{2} a^{3} \sin E}}{{R^{3} }} $$

using Eqs. (6), (9) and (10) in Eq. (7) yields

$$ \dot{\upsilon } = \frac{{n\left( {1 - e^{2} } \right)^{\frac{1}{2}} }}{{\left( {1 - e\cos E} \right)^{2} }};\;\; \ddot{\upsilon } = \frac{{ - 2en^{2} \left( {1 - e^{2} } \right)^{\frac{1}{2}} \sin E}}{{\rho^{4} }}\; {\text{and}} \;\rho = \frac{R}{a} $$

With the introduction of dimensionless pulsating coordinates using the following transformation by Singh and Umar^8^,

11 $$ X = \left( \frac{R}{a} \right)\xi , Y = \left( \frac{R}{a} \right)\eta , Z = \left( \frac{R}{a} \right)\zeta $$

Differentiating Eqs. (11) with $t$ and denoting the differentiation with respect to E by the prime, we obtain

$$ \dot{X} = \frac{ne\xi \sin E}{\rho } + n\xi^{\prime}, \dot{Y} = \frac{ne\eta \sin E}{\rho } + n\eta^{\prime}, \dot{Z} = \frac{ne\zeta \sin E}{\rho } + n\zeta^{\prime} $$

Also,

$$ \ddot{X} = \frac{{n^{2} \xi^{\prime\prime}}}{\rho } + \frac{{n^{2} e\xi^{\prime}\sin E}}{{\rho^{2} }} + \frac{{n^{2} e\xi \left( {\cos E - e} \right)}}{{\rho^{3} }} $$

$$ \ddot{Y} = \frac{{n^{2} \eta^{\prime\prime}}}{\rho } + \frac{{n^{2} e\eta^{\prime}\sin E}}{{\rho^{2} }} + \frac{{n^{2} e\eta \left( {\cos E - e} \right)}}{{\rho^{3} }} $$

$$ \ddot{Z} = \frac{{n^{2} \zeta^{\prime\prime}}}{\rho } + \frac{{n^{2} e\zeta^{\prime}\sin E}}{{\rho^{2} }} + \frac{{n^{2} e\zeta \left( {\cos E - e} \right)}}{{\rho^{3} }} $$

Considering the values of $X, \dot{X},\ddot{X}, Y, \dot{Y}, \ddot{Y}, \ddot{Z}, \dot{\upsilon } \;\;{\text{and}}\;\;\ddot{\upsilon }$ in Eq. (3) we obtain

12 $$ \begin{gathered} \xi^{\prime\prime} + \frac{{e\xi^{\prime}\sin E}}{\rho } - \frac{\xi }{\rho } - \frac{{2\left( {1 - e^{2} } \right)^{\frac{1}{2}} \eta^{\prime}}}{\rho } = \frac{\rho }{{n^{2} }}\frac{\partial U}{{\partial X}} \hfill \\ \eta^{\prime\prime} + \frac{{e\eta^{\prime}\sin E}}{\rho } - \frac{\eta }{\rho } - \frac{{2\left( {1 - e^{2} } \right)^{\frac{1}{2}} \xi^{\prime}}}{\rho } = \frac{\rho }{{n^{2} }}\frac{\partial U}{{\partial Y}} \hfill \\ \zeta^{\prime\prime} + \frac{{e\zeta^{\prime}\sin E}}{\rho } + \frac{{e\zeta \left( {\cos E - e} \right)}}{{\rho^{2} }} = \frac{\rho }{{n^{2} }}\frac{\partial U}{{\partial Z}} \hfill \\ \end{gathered} $$

Transforming $U$ in terms of the new coordinate system $\left( {\xi , \eta , \zeta } \right)$ denoted by $\overline{U}$, we have

$$ \overline{U} = k^{2} \left( {\frac{{m_{1} }}{{r_{1} }} + \frac{{m_{2} }}{{r_{2} }}} \right) $$

$$ r_{i}^{2} = \left( {\xi - \xi_{i} } \right)^{2} + \eta^{2} + \zeta^{2} , \left( {i = 1,2} \right) $$

13 $$ \xi_{1} = \frac{{ - m_{2} a}}{{M_{1} + M_{2} }}, \xi_{2} = \frac{{m_{1} a}}{{M_{1} + M_{2} }} $$

$$ \frac{\partial U}{{\partial X}} = \frac{1}{{\rho^{2} }}\frac{{\partial \overline{U}}}{\partial \xi }, \frac{\partial U}{{\partial Y}} = \frac{1}{{\rho^{2} }}\frac{{\partial \overline{U}}}{\partial \eta }, \frac{\partial U}{{\partial Z}} = \frac{1}{{\rho^{2} }}\frac{{\partial \overline{U}}}{\partial \zeta } $$

Comparing Eqs. (12) and (13) we obtain

14 $$ \begin{aligned} \xi^{\prime\prime} + \frac{{e\xi^{\prime}\sin E}}{\rho } - \frac{{2\left( {1 - e^{2} } \right)^{\frac{1}{2}} \eta^{\prime}}}{\rho } & = \frac{{\partial {\Omega }}}{\partial \xi } \\ \eta^{\prime\prime} + \frac{{e\eta^{\prime}\sin E}}{\rho } + \frac{{2\left( {1 - e^{2} } \right)^{\frac{1}{2}} \xi^{\prime}}}{\rho } & = \frac{{\partial {\Omega }}}{\partial \eta } \\ \zeta^{\prime\prime} + \frac{{e\zeta^{\prime}\sin E}}{\rho } & = \frac{{\partial {\Omega }}}{\partial \zeta } \\ \end{aligned} $$

With the force function

15 $$ {\Omega } = \frac{1}{{n^{2} \rho }}\left[ {\frac{{n^{2} }}{2}\left( {\xi^{2} + \eta^{2} } \right) - \frac{{en^{2} }}{2\rho }\left( {\cos E - e} \right)\zeta^{2} + \overline{U}} \right] $$

It is observed from Eq. (15) that the force function ${\Omega }$ depends on the eccentric anomaly $E$, that is it varies for different positions of the primaries. We consider the average force function ${\overline{\Omega }}$ as;

16 $$ {\overline{\Omega }} = \frac{1}{2\pi }\mathop \smallint \limits_{0}^{2\pi } {\Omega }dE = \frac{1}{{\left( {1 - e^{2} } \right)^{\frac{1}{2}} }}\left[ {\frac{1}{2}\left( {\xi^{2} + \eta^{2} } \right) + \frac{{\overline{U}}}{{n^{2} }}} \right] $$

Now, integrating Eqs. (14) With respect to $E$ and averaging, the system of equations reduces to the following;

17 $$ \begin{aligned} \xi^{\prime\prime} - 2\eta^{\prime} & = \frac{{\partial {\overline{\Omega }}}}{\partial \xi } \\ \eta^{\prime\prime} + 2\xi^{\prime} & = \frac{{\partial {\overline{\Omega }}}}{\partial \eta } \\ \zeta^{\prime\prime} & = \frac{{\partial {\overline{\Omega }}}}{\partial \zeta } \\ \end{aligned} $$

With the force function

18 $$ {\overline{\Omega }} = \frac{1}{{\left( {1 - e^{2} } \right)^{\frac{1}{2}} }}\left[ {\frac{1}{2}\left( {\xi^{2} + \eta^{2} } \right) + \frac{{\overline{U}}}{{n^{2} }}} \right] $$

Equations (17) and (18) represent the equations of motion of the general elliptic restricted three-body problem (ER3BP).

### Force due to radiation

Since the radiation pressure force, $F_{p}$ changes with distance by the law as the gravitational attraction force $F_{g}$ and acts opposite to it, it is possible that this force may lead to a reduction of the mass of the massive particle. Since this reduction of mass depends on the properties of the particles, then the resulting force on the particle is $F_{g} - F_{p} = F_{g} \left( {1 - \frac{{F_{p} }}{{F_{g} }}} \right) = qF_{g}$.

Where $q = 1 - \frac{{F_{p} }}{{F_{g} }}$ is the mass reduction factor.

We denote the radiation factor as $q_{i} \left( {i = 1,2} \right)$ for both primaries such that $0 < 1 - q_{i} \ll 1$, hence, instead of mass $m_{1}$, $m_{1} q_{1}$ will appear on the force function, and instead of $m_{2}$, $m_{2} q_{2}$ will appear on the force function.

### Force due to oblateness

According to McCuskey^42^, the force due to oblateness of the bigger primary with mass ${m}_{1}$ and smaller primary $m_{2}$ is given by

19 $$ F = \frac{{k^{2} m_{1} }}{{ r_{1}^{2} }} + \frac{{3k^{2} }}{{2 r_{1}^{4} }}\left( {\frac{{AE^{2} - AP^{2} }}{{5R^{2} }}} \right)m_{1} + \frac{{k^{2} m_{2} }}{{ r_{2}^{2} }} + \frac{{3k^{2} }}{{2 r_{2}^{4} }}\left( {\frac{{AE^{2} - AP^{2} }}{{5R^{2} }}} \right)m_{2} $$

where $AE$ and $AP$ are respectively the dimensional equatorial and polar radii of the primaries. Let ${\overline{\text{F}}}$ be the potential due to oblateness, then;

20 $$ F = \frac{{ - \partial {\overline{\text{F}}}}}{{\partial r_{1} }} + \frac{{ - \partial {\overline{\text{F}}}}}{{\partial r_{2} }} $$

Comparing Eqs. (19) and  (20) and then integrating with respect to $r_{1}$ and $r_{2}$ we have

$$ {\overline{\text{W}}} = k^{2} \left( {\frac{{m_{1} }}{{r_{1} }} + \frac{{A_{1} m_{1} }}{{2r_{1}^{3} }} + \frac{{m_{2} }}{{r_{2} }} + \frac{{A_{2} m_{2} }}{{2r_{2}^{3} }}} \right)\;\;{\text{with}}\;\;A_{i} = \frac{{A_{i} E^{2} - A_{i} P^{2} }}{{5R^{2} }}\left( {i = 1,2} \right). $$

Therefore, the force function due to the combined effects of oblateness and radiation on both primaries becomes;

21 $$ {\Omega } = \frac{1}{{\left( {1 - e^{2} } \right)^{\frac{1}{2}} }}\left[ {\frac{1}{2}\left( {\xi^{2} + \eta^{2} } \right) + \frac{{k^{2} }}{{n^{2} }}\left( {\frac{{m_{1} q_{1} }}{{r_{1} }} + \frac{{A_{1} m_{1} q_{1} }}{{2r_{1}^{3} }} + \frac{{m_{2} q_{2} }}{{r_{2} }} + \frac{{A_{2} m_{2} q_{2} }}{{2r_{2}^{3} }}} \right)} \right] $$

### Mean motion

According to Singh and Umar^8^, the distance between the primaries in the elliptic case is given as; $r = \frac{{a\left( {1 - e^{2} } \right)}}{1 + e\cos \mu }$ and the mean distance between them is given by

$$ \frac{1}{2\pi }\mathop \smallint \limits_{0}^{2\pi } {\text{r}}d\mu = \frac{{a\left( {1 - e^{2} } \right)}}{{\left( {1 + e^{2} } \right)^{\frac{1}{2}} }} $$

Szebehely^45^ asserted that the orbits of $m_{1}$ and $m_{2}$ with respect to the center of mass, with semi-major axes $a_{1} = am_{1}$ and $a_{2} = am_{2}$ respectively have the same eccentricity in the two-body problem. That is;

22 $$ \frac{{n^{2} aM_{1} \left( {1 - e^{2} } \right)}}{{\left( {1 + e^{2} } \right)^{\frac{1}{2}} }} = k^{2} M_{2} \left( {1 + \frac{{3A_{1} }}{2} + \frac{{3A_{2} }}{2}} \right)\;\;{\text{and}}\;\;\frac{{n^{2} aM_{2} \left( {1 - e^{2} } \right)}}{{\left( {1 + e^{2} } \right)^{\frac{1}{2}} }} = k^{2} M_{1} \left( {1 + \frac{{3A_{1} }}{2} + \frac{{3A_{2} }}{2}} \right) $$

Adding the Eqs. (22) we have

23 $$ n^{2} = \frac{{k^{2} \left( {1 + e^{2} } \right)^{\frac{1}{2}} }}{{a\left( {1 - e^{2} } \right)}}\left( {1 + \frac{{3A_{1} }}{2} + \frac{{3A_{2} }}{2}} \right) $$

Now, we choose the unit of time such as to make $k^{2} = 1$ and the unit of mass such that the sum of the mass of the finite bodies is taken as unity. For this, we take $m_{1} + m_{2} = 1$ where ${m}_{2}=\mu$.$0 < \mu = \frac{{m_{2} }}{{m_{1} + m_{2} }} < \frac{1}{2}$ and $1 - \mu = \frac{{m_{1} }}{{m_{1} + m_{2} }}$ such that $m_{1} > m_{2}$ are the masses of the bigger and smaller primaries and their coordinates are and $(1 - \mu ,0,0)$ respectively.$A_{i} < < 1$ (i = 1,2) depict the oblateness of the bigger ($A_{1}$) and smaller ($A_{2}$) primaries,${\text{ q}}_{1} ,{\text{ q}}_{2}$ are the radiation factors for both primaries; a is the semi-major axis of the orbit; $r_{i}$(i = 1,2) are their distances from the infinitesimal mass; e is the eccentricity of the orbits Where $\mu$ is the mass ratio. Then $m_{1} = 1 - \mu$. Also, $\xi_{1} = - \mu$ and $\xi_{2} = 1 - \mu$. Considering these terms in Eqs. (17), (18), (21) and (23) we obtain the equations of motion in the elliptic restricted three-body problem with oblate and radiating primaries as;

24 $$ \xi^{\prime\prime} - 2\eta^{\prime} = \frac{\partial \Omega }{{\partial \xi }} = \Omega_{\xi } , \eta^{\prime\prime} + 2\xi^{\prime} = \frac{\partial \Omega }{{\partial \eta }} = \Omega_{\xi } , \zeta^{\prime\prime} = \frac{\partial \Omega }{{\partial \zeta }} = \Omega_{\xi } $$

with the force function

25 $$ \Omega = \left( {1 - e^{2} } \right)^{{ - \frac{1}{2}}} \left[ {\frac{1}{2}\left( {\xi^{2} + \eta^{2} } \right) + \frac{1}{{n^{2} }}\left\{ {\frac{{\left( {1 - \mu } \right)q_{1} }}{{r_{1} }} + \frac{{\left( {1 - \mu } \right)A_{1} q_{1} }}{{2r_{1}^{3} }} + \frac{{\mu q_{2} }}{{r_{2} }} + \frac{{\mu A_{2} q_{2} }}{{2r_{2}^{3} }}} \right\}} \right] $$

The mean motion, $n,$ is given as

26 $$ n^{2} = \frac{{\left( {1 + e^{2} } \right)^{\frac{1}{2}} }}{{a\left( {1 - e^{2} } \right)}}\left[ {1 + \frac{{3A_{1} }}{2} + \frac{{3A_{2} }}{2}} \right] $$

27 $$ r_{i}^{2} = \left( {\xi - \xi_{i} } \right)^{2} + \eta^{2} + \zeta^{2} \;\;\left( {i = 1,2} \right) $$

### Force due to Poynting Robertson drag

Following Singh and Amuda^39^, the components of Poynting-Robertson(P-R) drag of the bigger and smaller primaries can be written as;

$$ \begin{aligned} &  \varepsilon - {\text{component}};\;\frac{{W_{1} }}{{r_{1}^{2} }}\left[ {\frac{{\left( {\xi + \mu } \right)}}{{r_{1}^{2} }}\left( {\mathop \xi \limits^{\prime } \left( {\xi + \mu } \right) + \eta \mathop \eta \limits^{\prime } + \zeta \mathop \zeta \limits^{\prime } } \right) + \mathop \xi \limits^{\prime } - n\eta } \right] \\ & \quad - \frac{{W_{2} }}{{r_{2}^{2} }}\left[ {\frac{{\left( {\xi + \mu - 1} \right)}}{{r_{2}^{2} }}\left( {\mathop \xi \limits^{\prime } \left( {\xi + \mu - 1} \right) + \eta \mathop \eta \limits^{\prime } + \zeta \mathop \zeta \limits^{\prime } } \right) + \mathop \xi \limits^{\prime } - n\eta } \right] \end{aligned} $$

$$ \begin{aligned}  & \eta {\text{ - Component}};\;\;\frac{{W_{1} }}{{r_{1}^{2} }}\left[ {\frac{\eta }{{r_{1}^{2} }}\left( {\mathop \xi \limits^{\prime } \left( {\xi + \mu } \right) + \eta \mathop \eta \limits^{\prime } + \zeta \mathop \zeta \limits^{\prime } } \right) + \mathop \eta \limits^{\prime } + n\left( {\xi + \mu } \right)} \right] \\ & \quad - \frac{{W_{2} }}{{r_{2}^{2} }}\left[ {\frac{\eta }{{r_{2}^{2} }}\left( {\mathop \xi \limits^{\prime } \left( {\xi + \mu - 1} \right) + \eta \mathop \eta \limits^{\prime } + \zeta \mathop \zeta \limits^{\prime } } \right) + \mathop \eta \limits^{\prime } + n\left( {\xi + \mu - 1} \right)} \right] \end{aligned} $$

28 $$ \zeta - {\text{Component}};\;\;\frac{{W_{1} }}{{r_{1}^{2} }}\left[ {\frac{\zeta }{{r_{1}^{2} }}\left( {\mathop \xi \limits^{\prime } \left( {\xi + \mu } \right) + \eta \mathop \eta \limits^{\prime } + \zeta \mathop \zeta \limits^{\prime } } \right) + \mathop \zeta \limits^{\prime } } \right] - \frac{{W_{2} }}{{r_{2}^{2} }}\left[ {\frac{\zeta }{{r_{2}^{2} }}\left( {\mathop \xi \limits^{\prime } \left( {\xi + \mu - 1} \right) + \eta \mathop \eta \limits^{\prime } + \zeta \mathop \zeta \limits^{\prime } } \right) + \mathop \zeta \limits^{\prime } } \right] $$

Simplifying Eq. (28), the equations of motion of the infinitesimal mass (test particle) in the elliptic restricted three-body problem when both primaries are oblate and luminous with Poynting-Robertson drag can be written using the dimensionless variables and a barycentric synodic coordinate system ($\xi$,$\eta$, $\zeta$) as:

29 $$ \xi^{\prime\prime} - 2\eta^{\prime} = \Omega_{\xi } \;\;\eta^{\prime\prime} + 2\xi^{\prime} = \Omega_{\xi } \;\; \zeta^{\prime\prime} = \Omega_{\xi } $$

where primes denote differentiation with respect to the eccentric anomaly.

$$ \begin{aligned}{\Omega }_{\xi }& =\left(1-{e}^{2}\right)\left\{{n}^{2}\xi -\frac{\left(1-\mu \right)\left(\xi +\mu \right){q}_{1}}{{r}_{1}^{3}}-\frac{\mu \left(\xi +\mu -1\right){q}_{2}}{{r}_{2}^{3}}-\frac{3\left(1-\mu \right)\left(\xi +\mu \right){A}_{1}{q}_{1}}{{2r}_{1}^{5}} \right. \\ & \quad  \left.  -\frac{3\mu \left(\xi +\mu -1\right){{A}_{2}q}_{2}}{2{r}_{2}^{5}}- \frac{{W}_{1}}{{r}_{1}^{2}}\left[\frac{\left(\xi +\mu \right)}{{r}_{1}^{2}}\left({\xi }{\prime}\left(\xi +\mu \right)+\eta {\eta }{\prime}+\zeta {\zeta }{\prime}\right)+{\xi }{\prime}-n\eta \right]\right. \\ & \quad  \left. -\frac{{W}_{2}}{{r}_{2}^{2}}\left[\frac{\left(\xi +\mu -1\right)}{{r}_{2}^{2}}\left({\xi }{\prime}\left(\xi +\mu -1\right)+\eta {\eta }{\prime}+\zeta {\zeta }{\prime}\right)+{\xi }{\prime}-n\eta \right]\right\}\end{aligned} $$

$$ \begin{aligned} \Omega_{\eta } & = \left( {1 - e^{2} } \right)\left\{ {n^{2} \eta - \frac{{\left( {1 - \mu } \right)q_{1} \eta }}{{r_{1}^{3} }} - \frac{{\mu q_{2} \eta }}{{r_{2}^{3} }} - \frac{{3\left( {1 - \mu } \right)A_{1} q_{1} \eta }}{{2r_{1}^{5} }} - \frac{{3\mu A_{2} q_{2} \eta }}{{2r_{2}^{5} }} }\right.\\ & \quad \left. {- \frac{{W_{1} }}{{r_{1}^{2} }}\left[ {\frac{\eta }{{r_{1}^{2} }}\left( {\xi^{\prime}\left( {\xi + \mu } \right) + \eta \eta^{\prime} + \zeta \zeta^{\prime}} \right) + \eta^{\prime} + n\left( {\xi + \mu } \right)} \right] }\right.\\ & \quad \left. { - \frac{{W_{2} }}{{r_{2}^{2} }}\left[ {\frac{\eta }{{r_{2}^{2} }}\left( {\xi^{\prime}\left( {\xi + \mu - 1} \right) + \eta \eta^{\prime} + \zeta \zeta^{\prime}} \right) + \eta^{\prime} + n\left( {\xi + \mu - 1} \right)} \right]} \right\} \end{aligned} $$

30 $$ \begin{aligned} \Omega_{\zeta } & = \frac{{\left( {1 - e^{2} } \right)}}{{n^{2} }}\left\{ { - \frac{{\left( {1 - \mu } \right)q_{1} \zeta }}{{r_{1}^{3} }} - \frac{{\mu q_{2} \zeta }}{{r_{2}^{3} }} - \frac{{3\left( {1 - \mu } \right)A_{1} q_{1} \zeta }}{{2r_{1}^{5} }} - \frac{{3\mu A_{2} q_{2} \zeta }}{{2r_{2}^{5} }} }\right. \\ & \quad  \left.{- \frac{{W_{1} }}{{r_{1}^{2} }}\left[ {\frac{\zeta }{{r_{1}^{2} }}\left( {\xi^{\prime}\left( {\xi + \mu } \right) + \eta \eta^{\prime} + \zeta \zeta^{\prime}} \right) + \zeta^{\prime}} \right]  - \frac{{W_{2} }}{{r_{2}^{2} }}\left[ {\frac{\zeta }{{r_{2}^{2} }}\left( {\xi^{\prime}\left( {\xi + \mu - 1} \right) + \eta \eta^{\prime} + \zeta \zeta^{\prime}} \right) + \zeta^{\prime}} \right]} \right\} \end{aligned} $$

31 $$ r_{1}^{2} = \left( {\xi + \mu } \right)^{2} + \eta^{2} + \zeta^{2} ;\;\;r_{2}^{2} = \left( {\xi + \mu - 1} \right)^{2} + \eta^{2} + \zeta^{2} $$

$0 < W_{i} \ll 1, i = 1,2$ illustrate the Poynting-Robertson drags of the primaries and are written as;

$$ W_{1} = \frac{{\left( {1 - \mu } \right)\left( {1 - q_{1} } \right)}}{{c_{d} }}\;\;W_{2} = \frac{{\mu \left( {1 - q_{2} } \right)}}{{c_{d} }} $$

where C_d_ characterizes the dimensionless velocity of light and q is a factor known as the radiation effect (Radzievsky^32^).

Thus, these equations are affected by the oblateness, radiation pressure, semi-major axis, eccentricity, and Poynting-Robertson drags of the primaries.

## Location of triangular equilibrium points

The equilibrium points are those points at which the velocity and acceleration are zero. Therefore, these points are the solutions to equations of the system (29).

where $\xi^{\prime} = \xi^{\prime\prime} = \eta^{\prime} = \eta^{\prime\prime} = \zeta^{\prime} = \zeta^{\prime\prime} = 0$. This implies that they are solutions of equations

32 $$ {\Omega }_{\xi } = {\Omega }_{\eta } = {\Omega }_{\zeta } = 0 $$

From the last equation one can obtain $\zeta = 0$ This shows that the coplanar stationary or equilibrium points exist.

The location of triangular libration points is obtained by the solutions of the first two equations taking into consideration $\eta \ne 0$, $\zeta = 0$.

Thus, from Eq. (32) we have

$$ n^{2} \xi - \frac{{\left( {1 - \mu } \right)\left( {\xi + \mu } \right)q_{1} }}{{r_{1}^{3} }} - \frac{{\mu \left( {\xi + \mu - 1} \right)q_{2} }}{{r_{2}^{3} }} - \frac{{3\left( {1 - \mu } \right)\left( {\xi + \mu } \right)A_{1} q_{1} }}{{2r_{1}^{5} }} - \frac{{3\mu \left( {\xi + \mu - 1} \right)A_{2} q_{2} }}{{2r_{2}^{5} }} + \frac{{nW_{1} \eta }}{{r_{1}^{2} }} + \frac{{nW_{2} \eta }}{{r_{2}^{2} }} = 0 $$

33 $$ n^{2} \eta - \frac{{\left( {1 - \mu } \right)q_{1} \eta }}{{r_{1}^{3} }} - \frac{{\mu q_{2} \eta }}{{r_{2}^{3} }} - \frac{{3\left( {1 - \mu } \right)A_{1} q_{1} \eta }}{{2r_{1}^{5} }} - \frac{{3\mu A_{2} q_{2} \eta }}{{2r_{2}^{5} }} - \frac{{nW_{1} \left( {\xi + \mu } \right)}}{{r_{1}^{2} }} - \frac{{nW_{2} \left( {\xi + \mu - 1} \right)}}{{r_{2}^{2} }} = 0 $$

For a triangular point, the motion occurs in the orbital plane ($\xi \eta$), on solving the two Eq. (33), we obtain; $\left( {1 - \mu } \right)\eta \left( {n^{2} - \frac{{q_{1} }}{{r_{1}^{3} }} - \frac{{3A_{1} q_{1} }}{{2r_{1}^{5} }}} \right) = \frac{{nW_{1} }}{2} - nW_{2} - \frac{{nW_{1} }}{{2r_{1}^{2} }}\left( {r_{2}^{2} - 1} \right)$

34 $$ \mu \eta \left( {n^{2} - \frac{{q_{2} }}{{r_{2}^{3} }} - \frac{{3A_{2} q_{2} }}{{2r_{2}^{5} }}} \right) = nW_{1} + \frac{{nW_{2} }}{2} + \frac{{nW_{2} }}{{2r_{2}^{2} }}\left( {r_{1}^{2} - 1} \right) $$

Using Eq. (34) in the photogravitational elliptic case, that is, when oblateness and P-R drag are absent, $A_{1} = A_{2} = W_{1} = W_{2} = 0,$ then Eq. (34) reduces to

$$ \left( {n^{2} - \frac{{q_{1} }}{{r_{1}^{3} }}} \right) = 0,\;\;\left( {n^{2} - \frac{{q_{2} }}{{r_{2}^{3} }}} \right) = 0 $$

Which provide us

$$ r_{1} = \left( {\frac{{q_{1} }}{{n^{2} }}} \right)^{\frac{1}{3}} ,\;\;r_{2} = \left( {\frac{{q_{2} }}{{n^{2} }}} \right)^{\frac{1}{3}} $$

If we accept that both primaries are oblate and radiating with P-R drag, then $W_{1} \, \ne \,0,\,W_{2} \, \ne \,,0\,\,A_{i} \, \ne \,0$,$\,A_{i} \, < 1$, $i = 1,2$. Let $\varepsilon_{1}$ and $\varepsilon_{2}$ be the perturbations caused by the presence of radiation, oblateness, and P-R drag, then the values of $r_{1}$ and $r_{2}$ will change slightly by $\varepsilon_{1}$, $\varepsilon_{2}$ respectively so that

35 $$ r_{1} = \varepsilon_{1} + q_{1}^{\frac{1}{3}} \left( \frac{1}{n} \right)^{\frac{2}{3}} ,\;\;r_{2} = \varepsilon_{2} + q_{2}^{\frac{1}{3}} \left( \frac{1}{n} \right)^{\frac{2}{3}} , $$

where $$\left| {\varepsilon_{1} } \right|,\left| {\varepsilon_{2} } \right| < < 1$$.

On using only linear terms in $$ A_{1}$$,$$ A_{2} ,and e^{2}$$ and neglecting their products, Eq. (26) becomes;

$$ n^{2} = \frac{1}{a}\left( {1 + \frac{3}{2}A_{1} + \frac{3}{2}A_{2} + \frac{3}{2}e^{2} } \right) $$

If $A_{1}$, $A_{2}$ = 0, we get

36 $$ n^{2} = \frac{1}{a}\left( {1 + \frac{3}{2}e^{2} } \right) $$

Substituting the value of Eq. (35) in Eq. (36), we obtain

37 $$ r_{1} = \left( {aq_{1} } \right)^{\frac{1}{3}} \left( {1 - \frac{{e^{2} }}{2}} \right) + \varepsilon_{1} \;\;r_{2} = \left( {aq_{2} } \right)^{\frac{1}{3}} \left( {1 - \frac{{e^{2} }}{2}} \right) + \varepsilon_{2} $$

Using equations. (36) and (37) in Eq. (34) and neglecting second and higher-order terms of small quantities; $e^{2} ,\left| {A_{1} } \right|,\left| {A_{2} } \right|,\left| {\varepsilon_{1} } \right|,\left| {\varepsilon_{2} } \right|,\left| {W_{1} } \right|\left| {W_{2} } \right|$, we get

$$ \varepsilon_{1} = - \frac{{\left( {aq_{1} } \right)^{\frac{1}{3}} }}{2}\left( {A_{1} + A_{2} - A_{1} \left( {aq_{1} } \right)^{{ - \frac{2}{3}}} + \frac{{naW_{1} }}{3(1 - \mu )\sqrt 3 } + \frac{{2naW_{2} }}{3(1 - \mu )\sqrt 3 }} \right) $$

38 $$ \varepsilon_{2} = - \frac{{\left( {aq_{2} } \right)^{\frac{1}{3}} }}{2}\left( {A_{1} + A_{2} - A_{2} \left( {aq_{2} } \right)^{{ - \frac{2}{3}}} - \frac{{2naW_{1} }}{3\mu \sqrt 3 } - \frac{{naW_{2} }}{3\mu \sqrt 3 }} \right) $$

Substituting the values of $\varepsilon_{1}$ and $\varepsilon_{2}$ from Eq. (38) in Eq. (37), we obtain;

$$ r_{1} = \left( {aq_{1} } \right)^{\frac{1}{3}} \left( {1 - \frac{{e^{2} }}{2}} \right) - \frac{{\left( {aq_{1} } \right)^{\frac{1}{3}} }}{2}\left( {A_{1} + A_{2} - A_{1} \left( {aq_{1} } \right)^{{ - \frac{2}{3}}} + \frac{{naW_{1} }}{3(1 - \mu )\sqrt 3 } + \frac{{2naW_{2} }}{3(1 - \mu )\sqrt 3 }} \right) $$

$$ r_{2} = \left( {aq_{2} } \right)^{\frac{1}{3}} \left( {1 - \frac{{e^{2} }}{2}} \right) - \frac{{\left( {aq_{2} } \right)^{\frac{1}{3}} }}{2}\left( {A_{1} + A_{2} - A_{2} \left( {aq_{2} } \right)^{{ - \frac{2}{3}}} - \frac{{2naW_{1} }}{3\mu \sqrt 3 } - \frac{{naW_{2} }}{3\mu \sqrt 3 }} \right) $$

then

$$ r_{1}^{2} = \left( {aq_{1} } \right)^{\frac{2}{3}} \left( {1 - e^{2} - A_{1} - A_{2} + A_{1} \left( {aq_{1} } \right)^{{ - \frac{2}{3}}} - \frac{{2naW_{1} }}{3(1 - \mu )\sqrt 3 } - \frac{{4naW_{2} }}{3(1 - \mu )\sqrt 3 }} \right) $$

39 $$ r_{2}^{2} = \left( {aq_{2} } \right)^{\frac{2}{3}} \left( {1 - e^{2} - A_{1} - A_{2} + A_{2} \left( {aq_{2} } \right)^{{ - \frac{2}{3}}} + \frac{{4naW_{1} }}{3\mu \sqrt 3 } + \frac{{2naW_{2} }}{3\mu \sqrt 3 }} \right) $$

Substituting values of $r_{1}^{2}$ and $r_{2}^{2}$ from Eq. (39) into Eq. (31) and then ignoring the second and higher order terms for $\xi$ and $\eta$, we obtain

$$ \begin{aligned} \xi & = \frac{1}{2} - \mu + \frac{{\left( {aq_{1} } \right)^{\frac{2}{3}} }}{2}\left( {1 - e^{2} - A_{1} - A_{2} + A_{1} \left( {aq_{1} } \right)^{{ - \frac{2}{3}}} - \frac{{2naW_{1} }}{3(1 - \mu )\sqrt 3 } - \frac{{4naW_{2} }}{3(1 - \mu )\sqrt 3 }} \right) \\ & \;\; - \frac{{\left( {aq_{2} } \right)^{\frac{2}{3}} }}{2}\left( {1 - e^{2} - A_{1} - A_{2} + A_{2} \left( {aq_{2} } \right)^{{ - \frac{2}{3}}} + \frac{{4naW_{1} }}{3\mu \sqrt 3 } + \frac{{2naW_{2} }}{3\mu \sqrt 3 }} \right) \\ \end{aligned} $$

$$ \begin{aligned} \eta^{2} & = - \frac{1}{4} + \frac{{\left( {aq_{1} } \right)^{{{\raise0.7ex\hbox{$2$} \!\mathord{\left/ {\vphantom {2 3}}\right.\kern-0pt} \!\lower0.7ex\hbox{$3$}}}} }}{2}\left( {1 - e^{2} - A_{1} - A_{2} + A_{1} \left( {aq_{1} } \right)^{{{\raise0.7ex\hbox{${ - 2}$} \!\mathord{\left/ {\vphantom {{ - 2} 3}}\right.\kern-0pt} \!\lower0.7ex\hbox{$3$}}}} - \frac{{2naW_{1} }}{3(1 - \mu )\sqrt 3 } - \frac{{4naW_{2} }}{3(1 - \mu )\sqrt 3 }} \right) \\ & \;\; + \frac{{\left( {aq_{2} } \right)^{{{\raise0.7ex\hbox{$2$} \!\mathord{\left/ {\vphantom {2 3}}\right.\kern-0pt} \!\lower0.7ex\hbox{$3$}}}} }}{2}\left( {1 - e^{2} - A_{1} - A_{2} + A_{2} \left( {aq_{2} } \right)^{{{\raise0.7ex\hbox{${ - 2}$} \!\mathord{\left/ {\vphantom {{ - 2} 3}}\right.\kern-0pt} \!\lower0.7ex\hbox{$3$}}}} + \frac{{4naW_{1} }}{3\mu \sqrt 3 } + \frac{{2naW_{2} }}{3\mu \sqrt 3 }} \right) \\ \end{aligned} $$

Substituting the value $$a = 1 - \alpha$$, $$q_{1} = 1 - \beta_{1}$$, $$q_{2} = 1 - \beta_{2}$$$$\alpha ,\beta_{1} ,\beta_{2} < < 1$$, in $$\xi$$ and $$\eta$$ working only with the linear terms in $$e^{2}$$,$$A_{1}$$,$$A_{2}$$,$$\alpha$$,$$\beta_{1}$$ and $$\beta_{2}$$, we obtain

40 $$ \xi = \frac{1}{2} - \mu - \frac{{\beta_{1} }}{3} + \frac{{\beta_{2} }}{3} + \frac{{A_{1} }}{2} - \frac{{A_{2} }}{2} - \frac{{nW_{1} \left( {2 - \mu } \right)}}{3\mu (1 - \mu )\sqrt 3 } - \frac{{nW_{2} \left( {1 + \mu } \right)}}{3\mu (1 - \mu )\sqrt 3 } $$

41 $$ \eta = \pm \frac{\sqrt 3 }{2}\left\{ {1 - \frac{4\alpha }{9} - \frac{{2\beta_{1} }}{9} - \frac{{2\beta_{2} }}{9} - \frac{{2e^{2} }}{3} - \frac{{A_{1} }}{3} - \frac{{A_{2} }}{3}} \right\} + \frac{{nW_{1} \left( {2 - 3\mu } \right)}}{9\mu (1 - \mu )} + \frac{{nW_{2} \left( {1 - 3\mu } \right)}}{9\mu (1 - \mu )} $$

The system of Eqs. (40) and (41) give the positions of triangular equilibrium points $L_{5}$ $(\xi , - \eta )$ respectively. The location or positions of $L_{4}$ $(\xi ,\eta )$ and $L_{5}$ $(\xi , - \eta )$ rely on the parameters stated; which are; mass ratio ($\mu$), oblateness ($A_{1} ,A_{2}$), radiation pressure ($q_{1} ,q_{2}$), semi-major axis ($a$), eccentricity ($e$), and P-R drag ($W_{1} ,W_{2} \,$) of both primaries.

## Linear stability of triangular equilibrium points

The stability of the test particle (infinitesimal mass) around an equilibrium point describes the coordinates of a given equilibrium point denoted by $(\xi_{0} ,\eta_{0} )$ and small displacements ($\sigma ,\omega$) from the point. So that

$$ \xi = \xi_{0} + \sigma \;\;{\text{and}}\;\;\eta = \eta_{0} + \omega , $$

Then the variational equations of motion are given as

42 $$ \begin{gathered} \ddot{\sigma } - 2\dot{\omega } = \sigma \Omega_{\xi \xi }^{0} + \omega \Omega_{\xi \eta }^{0} + \dot{\sigma }\Omega_{{\xi \dot{\xi }}}^{0} + \dot{\omega }\Omega_{{\xi \dot{\eta }}}^{0} \hfill \\ \ddot{\omega } + 2\dot{\sigma } = \omega \Omega_{\eta \eta }^{0} + \sigma \Omega_{\eta \xi }^{0} + \dot{\sigma }\Omega_{{\eta \dot{\xi }}}^{0} + \dot{\omega }\Omega_{{\eta \dot{\eta }}}^{0} \hfill \\ \end{gathered} $$

Then the characteristic equation that relates to (42) is given as

43 $$ \lambda^{4} + a_{1} \lambda^{3} + a_{2} \lambda^{2} + a_{3} \lambda + a_{4} = 0 $$

where

$$ a_{1} = - \Omega_{{\xi \dot{\xi }}}^{0} - \Omega_{{\eta \dot{\eta }}}^{0} $$

$$ a_{2} = 4 - \Omega^{o}_{\xi \xi } - \Omega^{0}_{\eta \eta } + \Omega_{{\xi \dot{\xi }}}^{0} \Omega_{{\eta \dot{\eta }}}^{0} - (\Omega_{{\xi \dot{\eta }}}^{0} )^{2} $$

44 $$ a_{3} = \Omega_{{\xi \dot{\xi }}}^{0} \Omega_{\eta \eta }^{0} + \Omega_{{\eta \dot{\eta }}}^{0} \Omega_{\xi \xi }^{0} + 2\Omega_{\xi \eta }^{0} - 2\Omega_{\eta \xi }^{0} - \Omega_{\xi \eta }^{0} \Omega_{{\eta \dot{\xi }}}^{0} - \Omega_{\eta \xi }^{0} \Omega_{{\xi \dot{\eta }}}^{0} $$

$$ a_{4} = \Omega_{\xi \xi }^{0} \Omega_{\eta \eta }^{0} - \Omega_{\xi \eta }^{0} \Omega_{\eta \xi }^{0} $$

Solving the second partial derivatives at the triangular libration point, we have

$$ \begin{aligned} \Omega^{0}_{\xi \xi } & = \frac{3}{4} + \frac{{9e^{2} }}{8} + \frac{\alpha }{2} + \left( {\frac{3\mu }{2} - \frac{1}{2}} \right)\beta_{1} + \left( {\frac{1}{2} - \frac{3\mu }{2}} \right)\beta_{2} + \left( {\frac{9}{4} - \frac{12\mu }{4}} \right)A_{1} \\ & \;\; - \left( {\frac{3}{4} - \frac{12\mu }{3}} \right)A_{2} - \frac{{(\mu^{2} - 13\mu + 8)}}{4\mu (1 - \mu )\sqrt 3 }W_{1} + \frac{{(\mu^{2} + 11\mu - 4)}}{4\mu (1 - \mu )\sqrt 3 }W_{2} \\ \end{aligned} $$

$$ \begin{aligned} \Omega_{\eta \eta }^{0} & = \frac{9}{4} + \frac{{3e^{2} }}{8} - \frac{\alpha }{2} + \left( {\frac{1}{2} - \frac{3\mu }{2}} \right)\beta_{1} - \left( {1 - \frac{3\mu }{2}} \right)\beta_{2} + \frac{3}{4}A_{1} + \frac{3}{4}A_{2} \\ & \;\;\; + \frac{{(5\mu^{2} - 17\mu + 8)}}{4\mu (1 - \mu )\sqrt 3 }W_{1} - \frac{{(5\mu^{2} + 7\mu - 4)}}{4\mu (1 - \mu )\sqrt 3 }W_{2} \\ \end{aligned} $$

$$ \begin{gathered} \Omega_{\xi \eta }^{0} = \frac{3\sqrt 3 }{2}\left\{ {\frac{1}{2} - \mu + \frac{{e^{2} }}{2} + \frac{{\left( {1 - 2\mu } \right)\alpha }}{3} - \frac{(1 + \mu )}{9}\beta_{1} + \frac{(2 - \mu )}{9}\beta_{2} + \left( {1 - \mu } \right)A_{1} - \mu A_{2} } \right\} \hfill \\ - \frac{{(27\mu^{2} - 31\mu + 8)}}{12\mu (1 - \mu )}W_{1} - \frac{{(27\mu^{2} - 23\mu + 4)}}{12\mu (1 - \mu )}W_{2} \hfill \\ \end{gathered} $$

$$ \Omega_{\xi \eta }^{0} = \Omega_{\eta \xi }^{0} $$

$$ \Omega_{{\xi \dot{\xi }}}^{0} = \frac{{ - 5W_{1} - 5W_{2} }}{4} $$

$$ \Omega_{{\eta \dot{\eta }}}^{0} = \frac{{ - 7W_{1} - 7W_{2} }}{4} $$

$$ \Omega_{{\xi \dot{\eta }}}^{0} = - \frac{{\sqrt 3 W_{1} }}{4} + \frac{{\sqrt 3 W_{2} }}{4} $$

$$ \Omega_{{\eta \dot{\xi }}}^{0} = - \frac{{\sqrt 3 W_{1} }}{4} + \frac{{\sqrt 3 W_{2} }}{4} $$

$$ \Omega_{{\xi \dot{\eta }}}^{0} = \Omega_{{\eta \dot{\xi }}}^{0} $$

45 $$ (\Omega_{{\xi \dot{\eta }}}^{0} )^{2} = 0 $$

Substituting the values $\Omega^{0}_{\xi \xi } ,\Omega^{0}_{\eta \eta } ,\Omega^{0}_{\xi \eta } ,\Omega^{0}_{\eta \xi } ,\Omega^{0}_{{\xi \dot{\xi }}} ,\Omega^{0}_{{\eta \dot{\eta }}} ,\Omega^{0}_{{\xi \dot{\eta }}} ,\Omega^{0}_{{\eta \dot{\xi }}}$ into Eq. (17), the system of equations becomes

$$ a_{1} = 3W_{1} + 3W_{2} $$

$$ a_{2} = 1 + 4\alpha + \frac{9}{2}e^{2} - \left( {\frac{3}{2} - 3\mu } \right)A_{1} + \left( {\frac{3}{2} - 3\mu } \right)A_{2} + \frac{{W_{1} }}{\sqrt 3 } - \frac{{W_{2} }}{\sqrt 3 } $$

$$ a_{3} = - 3W_{1} - \frac{21}{4}W_{2} - \frac{9\mu }{4}W_{1} + \frac{9\mu }{4}W_{2} $$

46 $$ \begin{aligned} a_{4} & = \frac{27\mu (1 - \mu )}{4} - \frac{9}{16}e^{2} - \frac{3}{2}(1 - 2\mu )\alpha + \frac{3}{2}\mu (1 - \mu )\beta_{1} + \frac{3}{2}\mu (1 - \mu )\beta_{2} \\ & \;\;\; + \frac{117}{4}\mu (1 - \mu )A_{1} + \frac{117}{4}\mu (1 - \mu )A_{2} + \frac{27( - 3\mu + 2)}{{4\sqrt 3 }}W_{1} + \frac{27( - 3\mu + 1)}{{4\sqrt 3 }}W_{2} \\ \end{aligned} $$

Now we wish to discuss analytically the stability of motion using the Routh- Hurwitz criterion^43–46^. For this, we first evaluate the principal diagonal minors of the Hurwitz matrix composed of the coefficients of polynomials (43). Substituting the values of $a_{1}$,$a_{2}$,$a_{3}$, and $a_{4}$ from Eq. (46) in Hurwitz diagonal minors, we have

$$ \Delta_{1} = a_{1} \;\;{\text{gives}} $$

$$ \Delta_{1} = 3W_{1} + 3W_{2} > 0 $$

$$ \Delta_{2} = a_{1} a_{2} - a_{3} \;\;{\text{gives}} $$

$$ \Delta_{2} = 6W_{1} + \frac{33}{4}W_{2} + \frac{9\mu }{4}W_{1} - \frac{9\mu }{4}W_{2} > 0 $$

$$ \Delta_{3} = a_{3} \left( {a_{1} a_{2} - a_{3} } \right) - a_{1}^{2} a_{4} \;\;{\text{gives}} $$

$$\begin{aligned}   \Delta _{3}  &  = \left( { - 3W_{1}  - \frac{{21}}{4}W_{2}  - \frac{{9\mu }}{4}W_{1}  + \frac{{9\mu }}{4}W_{2} } \right)\left( {6W_{1}  + \frac{{33}}{4}W_{2}  + \frac{{9\mu }}{4}W_{1}  - \frac{{9\mu }}{4}W_{2} } \right) \\     & \;\; - \left( {3W_{1}  + 3W_{2} } \right)^{2} \left[ {\frac{{27\mu \left( {1 - \mu } \right)}}{4} - \frac{9}{{16}}e^{2}  - \frac{3}{2}\left( {1 - 2\mu } \right)\alpha  + \frac{3}{2}\mu \left( {1 - \mu } \right)\beta _{1}  + \frac{3}{2}\mu \left( {1 - \mu } \right)\beta _{2} } \right. \\     & \;\;\;\left. { + \frac{{117}}{4}\mu \left( {1 - \mu } \right)A_{1}  + \frac{{117}}{4}\mu \left( {1 - \mu } \right)A_{2}  + \frac{{27\left( { - 3\mu  + 2} \right)}}{{4\sqrt 3 }}W_{1}  + \frac{{27\left( { - 3\mu  + 1} \right)}}{{4\sqrt 3 }}W_{2} } \right] \\  \end{aligned}$$

Which on neglecting higher order terms, reduces to

$$ \Delta_{3} = 0 $$

$$ \Delta_{4} = a_{4} \left[ {a_{3} \left( {a_{1} a_{2} - a_{3} } \right) - a_{1}^{2} a_{4} } \right] = a_{4} \Delta_{3} \;\;{\text{gives}} $$

$$ \Delta_{4} = 0\;\;\left( {{\text{Since}}\,\Delta_{3} = 0} \right) $$

Since Hurwitz minor matrices are not all greater than zero, therefore, by the Routh- Hurwitz criterion, the triangular points are unstable. Even the value of a_3_ shows that the triangular points are unstable.

## Numerical application

The triangular points can be obtained numerically. These are done for two binary systems 61 CYGNI and ARCHIRD, which have oblate and radiating primaries with Poynting Robertson drag. The numerical data relating to the system stated are shown in Table 1 below.

**Table 1:** Showing numerical compilation for the binary systems used. Sources: Singh and Ashagwu^6^ and Singh and Amuda^39^.

Binary system	Masses (MSUN)		Mass ratio	Luminosity (LSUN)		Radiation pressures		Binary separation (ASUN) Au	Dimensionless velocity (Cd)
	$m_{1}$	$m_{2}$		$L_{1}$	$L_{2}$	$q_{1}$	$q_{2}$		
61 Cygni	0.70	0.63	0.4737	0.153	0.085	0.999767	0.999856	84	92,221.4
Archird	0.95	0.62	0.3949	1.29	0.06	0.9971	0.9997	71	67,675.52

Using the value $q = 1 - \frac{A\kappa L}{{r\rho M}}$ by Stefan-Boltzmann’s law we compute the radiation pressure factor q by taking $\kappa = 1$, M as the mass of the star, L as the luminosity of a star, r as the radius, $\rho$ as the density of a moving body and $\kappa$ as the efficiency factor of a star; $A = \frac{3}{16\pi CG}$ is a constant. In the C.G.S. system, $A = 2.9838 \times 10^{ - 5}$, $r = 0.02cm$ and $\rho = 1.4gcm^{ - 3}$ for some dust grain particles in the systems. The mass ratio is $\mu = \frac{{m_{2} }}{{m_{1} + m_{2} }}$. The dimensionless velocity of light (C_d_) for the binary stars according to^39,47^ is computed as $C_{d} = \frac{c}{{\sqrt {\frac{{\gamma \left( {m_{1} + m_{2} } \right)}}{{A_{u} }}} }}$ Here A_u_ is the binary separation of the primaries, $c$ is the velocity of light, and $\gamma$ is the gravitational constant; $m_{1}$, $m_{2}$ are masses of the bigger and smaller primary respectively. In C.G.S. system, $c = 2.99792458 \times 10^{10} cms^{ - 1}$ and $\gamma = 6.6743 \times 10^{ - 8} cm^{2} g^{ - 1} s^{ - 2}$.

Using these data, we compute the locations of triangular points, showing the effects of the parameters on the binary systems 61 CYGNI and ARCHIRD. The effect of the oblateness of $A_{1}$ in the absence of $A_{2}$_,_ and then with a constant $A_{2}$ is shown in Tables 2 and 3 respectively. Then, in the absence of $A_{1}$ and keeping $A_{1}$ constant, the effect of $A_{2}$ is examined (Table 4 and 5 respectively) using the Mathematica 12.1 software. We show the effects of radiation pressure ($q$), eccentricity ($e$), and semi-major axis ($a$), on the triangular points (Tables 6, 7, 8, and 9) for arbitrary values.

**Table 2:** Showing different values of ${\varvec{A}}_{1}$ in the absence of ${\varvec{A}}_{2}$ on the triangular points of 61 CYGNI and ARCHIRD.

${{\varvec{A}}}_{1}$	${{\varvec{A}}}_{2}$	61 CYGNI		ARCHIRD	
		$\xi$	$\pm \eta$	$\xi$	$\pm \eta$
0	0	0.0262835	0.554253	0.104939	0.252572i
0.0001	0	0.0263335	0.554232	0.104988	0.252523i
0.001	0	0.0267835	0.554043	0.105439	0.252082i
0.01	0	0.0312837	0.552145	0.109941	0.247619i
0.1	0	0.0762856	0.532796	0.15496	0.197531i
0.2	0	0.126288	0.510438	0.204982	0.11935i

**Table 3:** Showing different values of ${{\varvec{A}}}_{1}$ with constant ${{\varvec{A}}}_{2}$ on the triangular points of 61 CYGNI and ARCHIRD.

$A_{1}$	$A_{2}$	61 CYGNI		ARCHIRD	
		$\xi$	$\pm \eta$	$\xi$	$\pm \eta$
0	0	0.0262835	0.554253	0.104939	0.252572i
0.0001	0.02	0.0163339	0.550007	0.0949928	0.242514i
0.001	0.02	0.0167839	0.549816	0.095443	0.242054i
0.01	0.02	0.0212841	0.547904	0.0999449	0.237404i
0.1	0.02	0.0662861	0.5248	0.144965	0.184564i
0.2	0.02	0.116288	0.505847	0.194987	0.0963819i

**Table 4:** Showing different values of ${{\varvec{A}}}_{2}$ in the absence of ${{\varvec{A}}}_{1}$ on the triangular points of 61 CYGNI and ARCHIRD.

$A_{1}$	$A_{2}$	61 CYGNI		ARCHIRD	
		$\xi$	$\pm \eta$	$\xi$	$\pm \eta$
0	0	0.0262835	0.554253	0.104939	0.252572i
0	0.0002	0.0261835	0.554211	0.104838	0.252474i
0	0.002	0.0252835	0.553832	0.103939	0.25159i
0	0.02	0.0162839	0.550029	0.0949427	0.242565i

**Table 5:** Showing different values of ${{\varvec{A}}}_{2}$ with constant ${{\varvec{A}}}_{1}$ on the triangular points of 61 CYGNI and ARCHIRD.

$A_{1}$	$A_{2}$	61 CYGNI		ARCHIRD	
		$\xi$	$\pm \eta$	$\xi$	$\pm \eta$
0.001	0	0.0267835	0.554043	0.104439	0.252082i
0.001	0.0002	0.0266835	0.554	0.105339	0.251983i
0.001	0.002	0.0257835	0.553621	0.104439	0.251097i
0.001	0.02	0.0167839	0.549816	0.095443	0.242054i

**Table 6:** Showing varying values of radiation pressure on the triangular points **with** $A_{1} = 0.02$, $A_{2} = 0.04$, $e = 0.8$, a $= 0.8$, $W_{1} = 2.159 \times 10^{ - 8}$, $W_{2} = 1.436 \times 10^{ - 10}$.

${q}_{1}$	${q}_{2}$	$\xi$	$\pm \eta$
0.89	0.93	− 0.027592	0.457569
0.90	0.94	− 0.027569	0.460988
0.91	0.95	− 0.027546	0.464369
0.92	0.96	− 0.027524	0.467714
0.93	0.97	− 0.027502	0.471023
0.94	0.98	− 0.027481	0.474298
0.95	0.99	− 0.027459	0.47754

**Table 7:** Showing varying values of eccentricity on the triangular points with $A_{1} = 0.02$, $A_{2} = 0.04$, $a = 0.8$, $q_{1} = 0.79$, $q_{2} = 0.89$, $W_{1} = 2.159 \times 10^{ - 8}$, $W_{2} = 1.436 \times 10^{ - 10}$.

${\varvec{e}}$	$\xi$	$\pm \eta$
0.88	− 0.025914	0.322207i
0.77	− 0.030970	0.153363
0.66	− 0.035352	0.365896
0.55	− 0.039060	0.476719
0.44	− 0.042094	0.551056
0.33	− 0.044454	0.602567
0.22	− 0.046139	0.636815
0.11	− 0.047150	0.656507

**Table 8:** Showing varying values of Semi major axis on the triangular points **with** $A_{1} = 0.02$, $A_{2} = 0.04$, $e = 0.8$, $q_{1} = 0.79$, $q_{2} = 0.89$, $W_{1} = 2.159 \times 10^{ - 8}$, $W_{2} = 1.436 \times 10^{ - 10}$.

$a$	$\xi$	$\pm \eta$
0.9	− 0.040405	0.511018
0.8	− 0.038963	0.474136
0.7	− 0.037458	0.432342
0.6	− 0.035880	0.383638
0.5	− 0.034275	0.324275
0.4	− 0.032427	0.245376
0.3	− 0.030485	0.106339
0.2	− 0.028309	0.208516i
0.1	− 0.025717	0.329847i

**Table 9:** Showing varying values of Poynting drag (P-R drag) on the triangular points **with** $A_{1} = 0.02$, $A_{2} = 0.04$, $e = 0.8$, $q_{1} = 0.79$, $q_{2} = 0.89$, $a = 0.8$

$W_{1}$	$W_{2}$	$\xi$	$\pm \eta$
$0$	$0$	− 0.037458	0.432342
$2.1259 \times 10^{ - 8}$	$0$	− 0.037458	0.432342
$1.4179 \times 10^{ - 9}$	$0$	− 0.037458	0.432342
$2.1519 \times 10^{ - 8}$	$1.1143 \times 10^{ - 9}$	− 0.037458	0.432342
$1.7543 \times 10^{ - 8}$	$4.1131 \times 10^{ - 10}$	− 0.037458	0.432342
$1.1143 \times 10^{ - 9}$	$6.2071 \times 10^{ - 10}$	− 0.037458	0.432342
$1.8743 \times 10^{ - 9}$	$8.3121 \times 10^{ - 10}$	− 0.037458	0.432342
$0$	$1.9859 \times 10^{ - 8}$	− 0.037458	0.432342
$0$	$7.1143 \times 10^{ - 9}$	− 0.037458	0.432342
$0$	$6.1098 \times 10^{ - 10}$	− 0.037458	0.432342

## Numerical investigation for stability of triangular equilibrium points

The roots of Eq. (43) are written as

$$ \lambda_{1} = - \frac{{a_{1} }}{4} - \frac{1}{2}\sqrt {\frac{{a_{1}^{2} }}{4} - \frac{{2a_{2} }}{3} + S + T} - \frac{1}{2}\left( {\frac{{a_{1}^{2} }}{2} - \frac{{4a_{2} }}{3} - S - T - \frac{{\left( { - a_{1}^{3} + 4a_{1} a_{2} - 8a_{3} } \right)}}{{\sqrt[4]{{\frac{{a_{1}^{2} }}{4} - \frac{{2a_{2} }}{3} + S + T}}}}} \right)^{\frac{1}{2}} $$

$$ \lambda_{2} = - \frac{{a_{1} }}{4} + \frac{1}{2}\sqrt {\frac{{a_{1}^{2} }}{4} - \frac{{2a_{2} }}{3} + S + T} - \frac{1}{2}\left( {\frac{{a_{1}^{2} }}{2} - \frac{{4a_{2} }}{3} - S - T - \frac{{\left( { - a_{1}^{3} + 4a_{1} a_{2} - 8a_{3} } \right)}}{{\sqrt[4]{{\frac{{a_{1}^{2} }}{4} - \frac{{2a_{2} }}{3} + S + T}}}}} \right)^{\frac{1}{2}} $$

$$ \lambda_{3} = - \frac{{a_{1} }}{4} - \frac{1}{2}\sqrt {\frac{{a_{1}^{2} }}{4} - \frac{{2a_{2} }}{3} + S + T} + \frac{1}{2}\left( {\frac{{a_{1}^{2} }}{2} - \frac{{4a_{2} }}{3} - S - T - \frac{{\left( { - a_{1}^{3} + 4a_{1} a_{2} - 8a_{3} } \right)}}{{\sqrt[4]{{\frac{{a_{1}^{2} }}{4} - \frac{{2a_{2} }}{3} + S + T}}}}} \right)^{\frac{1}{2}} $$

47 $$ \lambda_{4} = - \frac{{a_{1} }}{4} + \frac{1}{2}\sqrt {\frac{{a_{1}^{2} }}{4} - \frac{{2a_{2} }}{3} + S + T} + \frac{1}{2}\left( {\frac{{a_{1}^{2} }}{2} - \frac{{4a_{2} }}{3} - S - T - \frac{{\left( { - a_{1}^{3} + 4a_{1} a_{2} - 8a_{3} } \right)}}{{\sqrt[4]{{\frac{{a_{1}^{2} }}{4} - \frac{{2a_{2} }}{3} + S + T}}}}} \right)^{\frac{1}{2}} $$

where:

$$ S = \frac{{\left( {2^{{{1 \mathord{\left/ {\vphantom {1 3}} \right. \kern-0pt} 3}}} \left( {a_{2}^{2} - 3a_{1} a_{3} + 12a_{4} } \right)} \right)}}{{3\left( {\sqrt[3]{{\left( {2a_{2}^{3} - 9a_{1} a_{2} a_{3} + 27a_{3}^{2} + 27a_{1}^{2} a_{4} - 72a_{2} a_{4} + \sqrt {\left( { - 4\left( {a_{2}^{2} - 3a_{1} a_{3} + 12a_{4} } \right)^{3} + \left( {2a_{2}^{3} - 9a_{1} a_{2} a_{3} + 27a_{3}^{2} + 27a_{1}^{2} a_{4} - 72a_{2} a_{4} } \right)^{2} } \right)} } \right)}}} \right)}} $$

$$ T = \frac{{2a_{2}^{3} - 9a_{1} a_{2} a_{3} + 27a_{3}^{2} + 27a_{1}^{2} a_{4} - 72a_{2} a_{4} + \sqrt {\left( { - 4\left( {a_{2}^{2} - 3a_{1} a_{3} + 12a_{4} } \right)^{3} + \left( {2a_{2}^{3} - 9a_{1} a_{2} a_{3} + 27a_{3}^{2} + 27a_{1}^{2} a_{4} - 72a_{2} a_{4} } \right)^{2} } \right)} }}{{3\left( {\sqrt[3]{2}} \right)}} $$

The roots $\lambda_{i} \left( {i = 1,2,3,4} \right)$ of Eq. (47) are computed numerically using the software MATHEMATICA 12.1 for the binary stars in the tables below:

## Discussion

We have studied the effects of oblateness, radiation pressure, eccentricity, and semi-major axis of the orbits of primaries together with P-R drag, on the motion of a test particle in the neighbourhood of triangular points in the ER3BP described by Eqs. (29)–(31). Equations (40) and (41) give the positions of triangular points similar to that of ^6^ in the absence of zonal harmonics $J_{4}$ and Poynting Robertson (P-R) drag (for $B_{1} = B_{2} = W_{1} = W_{2} = 0$). This also validates the position of triangular points for other generalizations by suppressing certain parameters. In the absence of radiation pressure force, oblateness and P-R drag effects, it reduces to the classical case of Szebehely^48^, i.e., when the primaries are spherical and non-emitters of radiation and without P-R drag. The location or position of the triangular points is computed numerically and shown in Tables 2, 3, 4, 5, 6, 7, 8 and 9, with each figure drawn and highlighted on the corresponding table.

**Figure 2:**
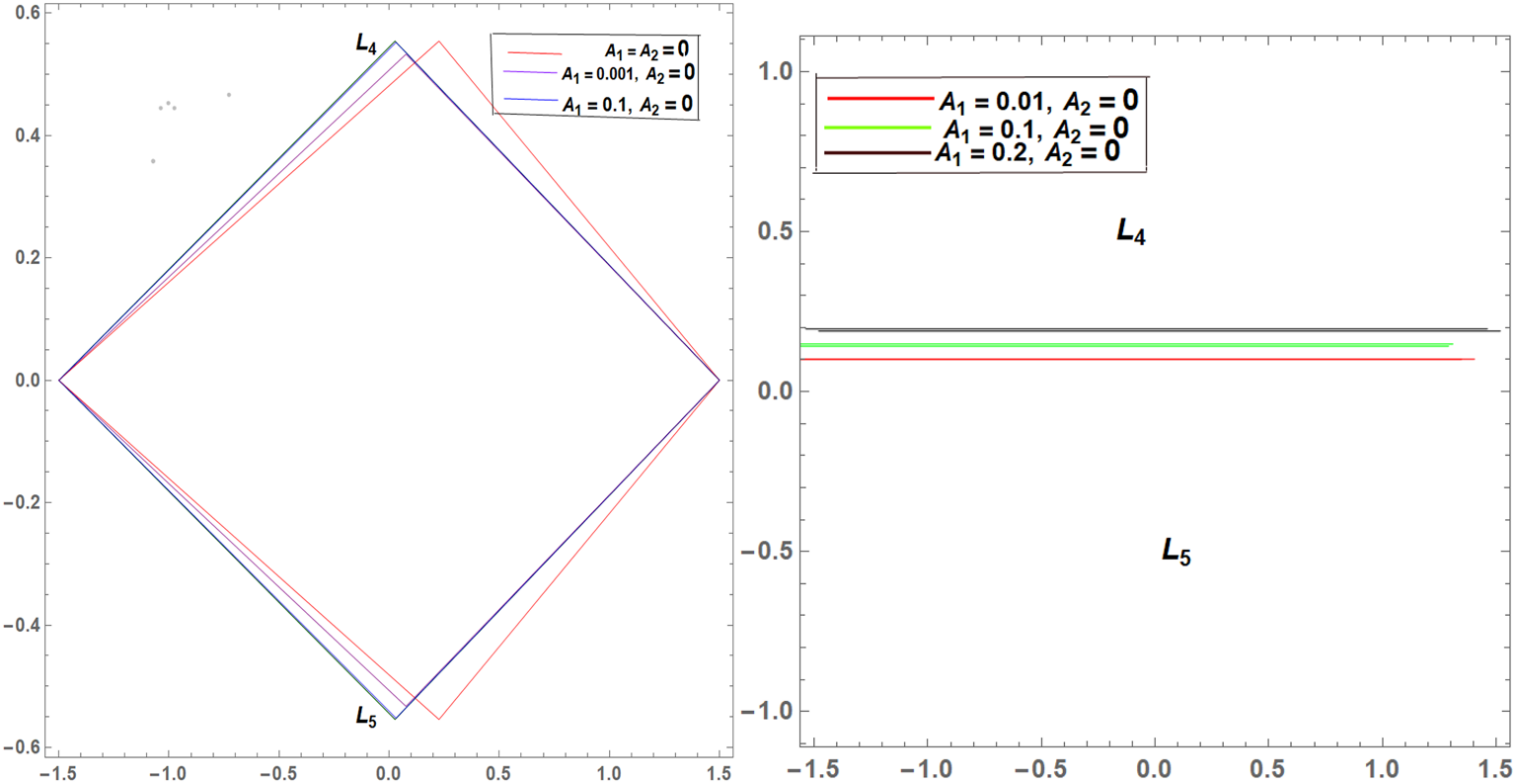
Showing the effect of different values of ${\varvec{A}}_{1}$ on the triangular points of 61 CYGNI (left diagram) and ARCHIRD (right diagram) in the absence of ${\varvec{A}}_{2}$.

**Figure 3:**
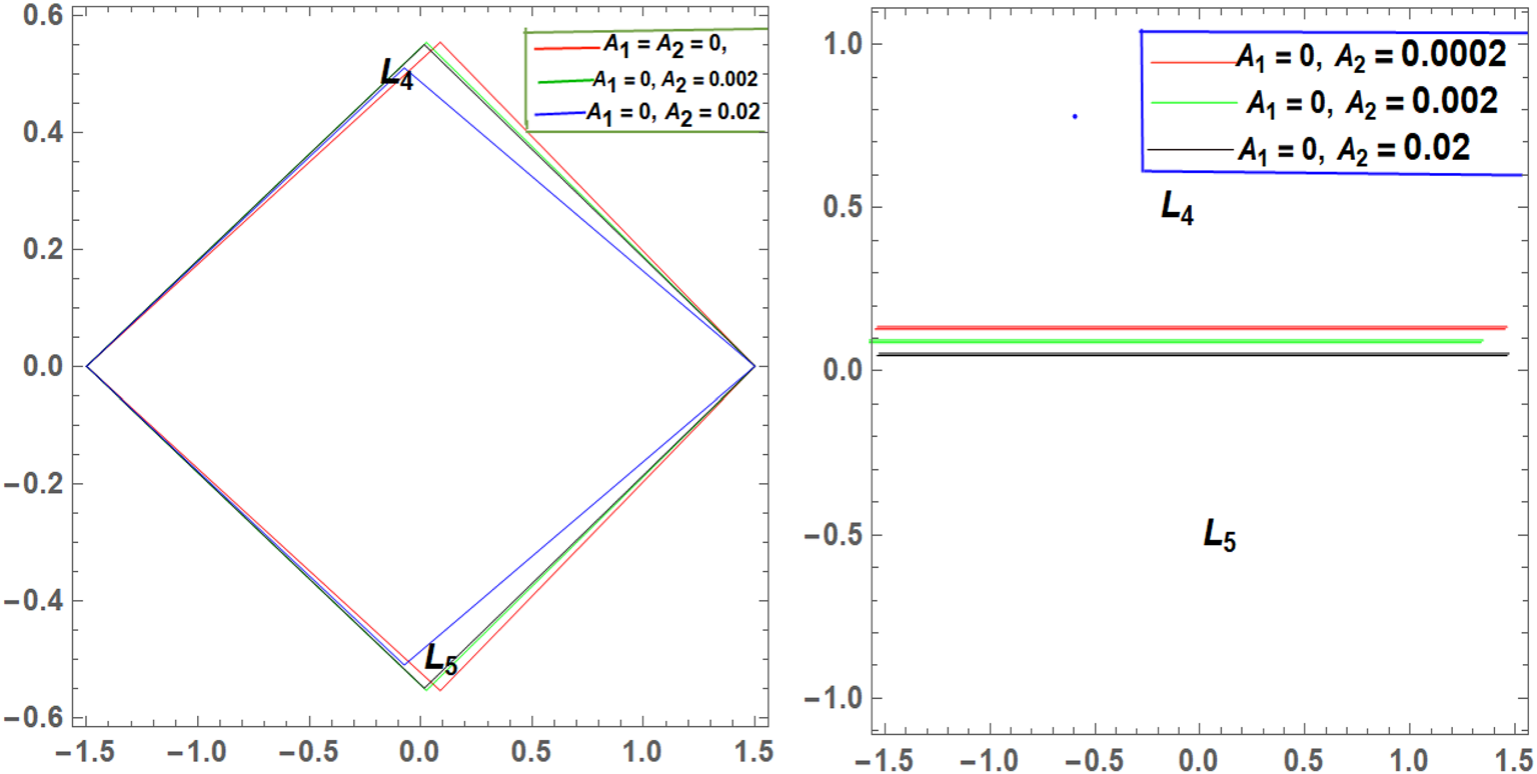
Showing the effect of different values of ${\varvec{A}}_{2}$ on the triangular points of 61 CYGNI (left diagram) and ARCHIRD (right diagram) in the absence of ${\varvec{A}}_{1}$.

**Figure 4:**
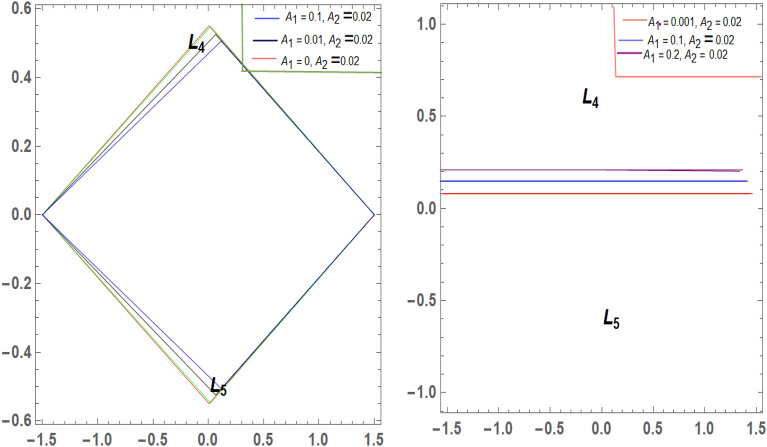
Showing the effect of different values of ${\varvec{A}}_{1}$ on the triangular points of 61 CYGNI (left diagram) and ARCHIRD (right diagram) with constant ${\varvec{A}}_{2}$.

**Figure 5:**
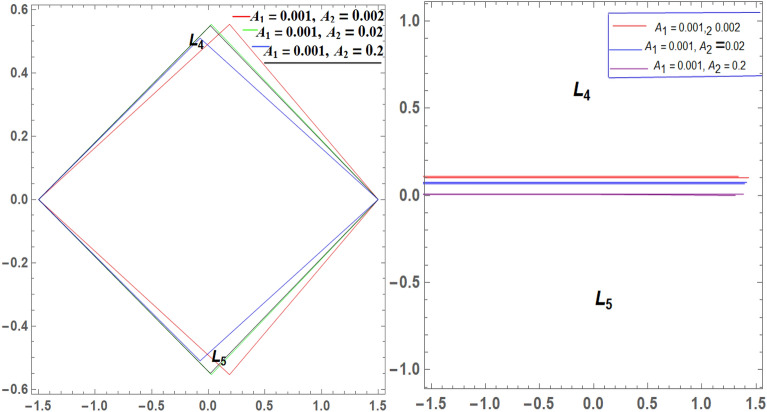
Showing the effect of different values of ${\varvec{A}}_{2}$ on the triangular points of 61 CYGNI and ARCHIRD with constant ${\varvec{A}}_{1}$.

**Figure 6:**
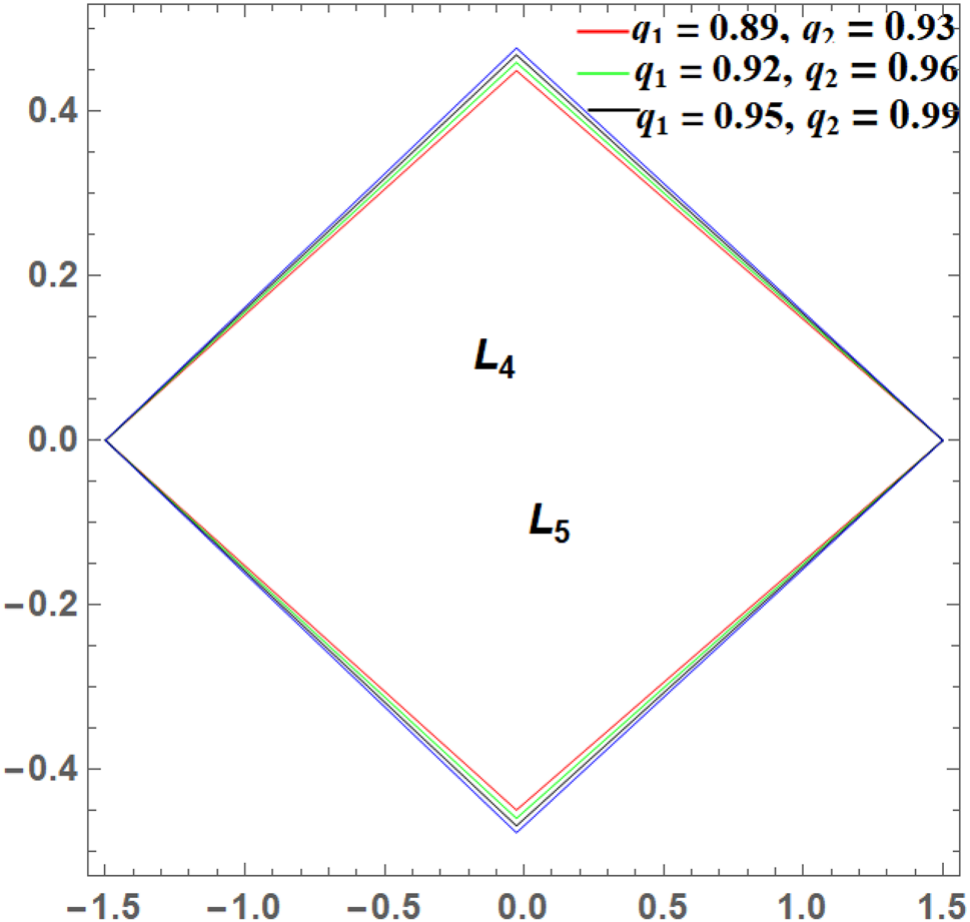
Showing the effect of different values of radiation pressure on the triangular points **with** ${A}_{1}=0.02, {A}_{2}$ = 0.04, $a = 0.8$,$e = 0.8$, $W_{1} = 2.159 \times 10^{ - 8}$& $$W_{2} = 1.436 \times 10^{\begin{subarray}{l} - 10 \\ \end{subarray} }$$.

**Figure 7:**
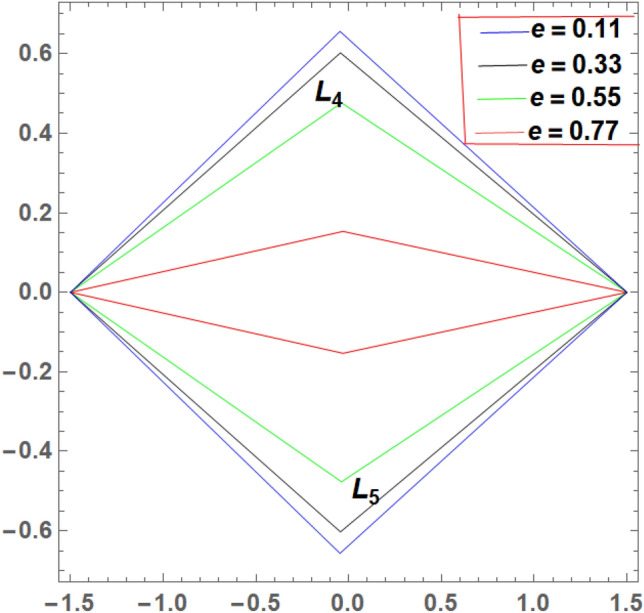
Showing the effect of different values of eccentricity on the triangular points with ${A}_{1}=0.02, {A}_{2}$ = 0.04, $a = 0.8$ $q_{1} = 0.79$,$q_{2} = 0.89$ $W_{1} = 2.159 \times 10^{ - 8}$ and $W_{2} = 1.436 \times 10^{ - 10}$.

**Figure 8:**
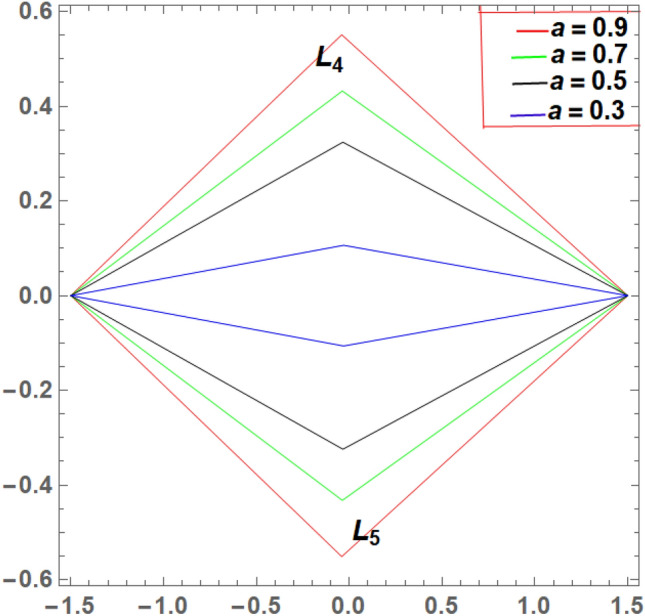
Showing the effect of different values of Semi major axis on the triangular points **with** ${A}_{1}=0.02, {A}_{2}$ = 0.04, $e = 0.8$ $q_{1} = 0.79$, $q_{2} = 0.89$ $W_{1} = 2.159 \times 10^{ - 8}$and $W_{2} = 1.436 \times 10^{ - 10}$.

**Figure 9:**
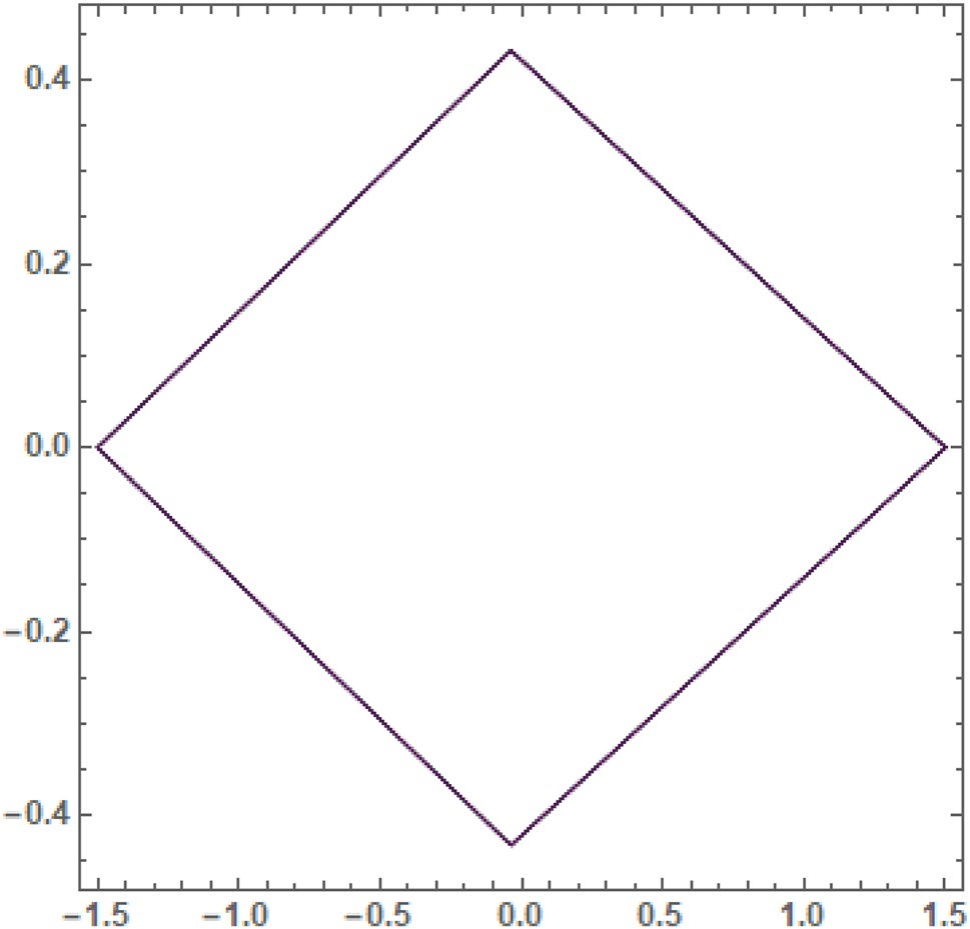
Showing the effect of different values of P-R drag on the triangular points **with** ${A}_{1}=0.02, {A}_{2}$ = 0.04, $q_{1} = 0.79$, $q_{2} = 0.89$,$e = 0.8$,$a = 0.8$

It is seen from Tables 2 and 3 that, $\xi$ increases and $\eta$ decreases with A_1_ in the case of 61 Cygni, while in the case of ARCHIRD, $\xi$ increases with A_1_ and $\eta$ does not exist. It converts into collinear movement through $\xi$ axis only as shown in Figs. 2, 3 and 4 respectively. In Tables 4 and 5, $\xi$ decreases, and $\eta$ decreases with A_2_ in the case of 61 Cygni, while in the case of ARCHIRD, it moves towards the origin along the $\xi$ axis only and $\eta$ does not exist. It converts into collinear points as shown in Figs. 3 and 5 respectively. Decreasing eccentricity while radiation pressure, oblateness, and P-R drag remain constant causes a shift away from the origin in the negative direction and away from the line joining the primaries. This is shown in Table 7 and Figs. 6, 7, $\xi$ increasing in the negative direction and $\eta$ moves away from $\xi$ axis. It can be seen that for high eccentricity (e = 0.88), the triangular points do not exist, thereby making it collinear. By comparing the present work with Singh and Amuda^39^, for circular restricted three-body problem (where e = 1) the triangular point ceases to exist. This is not the case with a decrease in the semi-major axis (Table 8 and Fig. 8). We also observe that, for a small value of the semi-major axis (a = 0.2), the triangular points cease to exist, thereby making it collinear. Also, increasing the radiation pressure with constant oblateness decreases $\xi$ towards the origin and $\eta$ increases away from the $\xi$ axis as shown in Table 6 and Fig. 6. A change in the position of the triangular equilibrium points due to P-R drag forces has been found and it was observed that the system causes no change in the position of triangular points (as shown in Table 9 and Fig. 9).

In analyzing the stability of the triangular equilibrium points analytically, we have applied the Routh-Hurwitz criterion. Since Hurwitz minor matrices are not all greater than zero, therefore, by Routh-Hurwitz criterion, the triangular equilibrium points are unstable. Particularly, when $A_{1} = A_{2} = W_{1} = W_{2} = e = \alpha = \beta_{1} = \beta_{2} = 0$, then $a_{1} = 0,a_{2} = 1,a_{3} = 0$ and $a_{4} = \frac{27}{4}\mu \left( {1 - \mu } \right)$ Szebehely^45^. This gives the classical case.

The roots of the characteristics Eq. (43) are given in Eq. (47) where values of $a_{1}$,$a_{2}$,$a_{3}$ and $a_{4}$ are provided in Eq. (46). These equations all depend on the parameters of the perturbing factors.

It is observed that the nature of the value $a_{3}$ as compared to the condition necessary for stability by Routh Hurwitz, shows that the motion of an infinitesimal mass (test particle) around triangular points is unstable.

The numerical values of the four roots of the characteristic Eq. (43) as given by Eq. (47) are shown in Tables 10, 11, 12. Tables 10 and 11 show that the system is stable in the presence of oblateness. But in the presence of both oblateness and P-R drag, it is unstable. Table 12, also shows that in the presence of radiation, the system is stable, but in the presence of PR-drag, whether radiation is present or absent, the system remains unstable.

**Table 10:** Showing the roots of characteristic equation for 61 CYGNI with different values of oblateness and P-R drag with q_1_ = 0.9997676, q_2_ = 0.999856, a = 0.628, and e = 0.4900.

A_1_	A_2_	W_1_	W_2_	Stability of triangular equilibrium points		Remark
				$\lambda_{1,2}$	$\lambda_{3,4}$	
$0$	$0$	$0$	$0$	$\mp 0.702793i$	$\mp 1.75343i$	Stable
		$1.32971 \times 10^{ - 9}$	$7.3966\,3 \times 10^{ - 10}$	$- 5.34529\, \times 10^{ - 9} \mp 1.75343i$	$2.24123\, \times 10^{ - 9} \mp 0.702793i$	Unstable
$0.0001$	$0.0003$	$0$	$0$	$\mp 0.703444i$	$\mp 1.75318i$	Stable
		$1.32971 \times 10^{ - 9}$	$7.3966\,3 \times 10^{ - 10}$	$- 5.34797 \times 10^{ - 9} \mp 1.75318i$	$2.24391 \times 10^{ - 9} \mp 0.70344i$	Unstable
$0.001$	$0.003$	$0$	$0$	$\mp 0.709298i$	$\mp 1.75086i$	Stable
		$1.32971 \times 10^{ - 9}$	$7.3966\,3 \times 10^{ - 10}$	$- 5.37235 \times 10^{ - 9} \mp 1.75086i$	$2.26829 \times 10^{ - 9} \mp 0.709298i$	Unstable
$0.01$	$0.03$	$0$	$0$	$\mp 0.767392i$	$\mp 1.7266i$	Stable
		$1.32971 \times 10^{ - 9}$	$7.3966\,3 \times 10^{ - 10}$	$- 5.645 \times 10^{ - 9} \mp 1.7266i$	$2.54094 \times 10^{ - 9} \mp 0.767392i$	Unstable
$0.01$	$0$	$0$	$0$	$\mp 0.707923i$	$\mp 1.75114i$	Stable
		$1.32971 \times 10^{ - 9}$	$7.3966\,3 \times 10^{ - 10}$	$- 5.36738 \times 10^{ - 9} \mp 1.751114i$	$2.26333 \times 10^{ - 9} \mp 0.707923i$	Unstable
$0.1$	$0$	$0$	$0$	$\mp 0.753958i$	$\mp 1.72977i$	Stable
		$1.32971 \times 10^{ - 9}$	$7.3966\,3 \times 10^{ - 10}$	$- 5.589 \times 10^{ - 9} \mp 1.72977i$	$2.48184 \times 10^{ - 9} \mp 0.753958i$	Unstable
	$0$	$0$	$0$	$\mp 0.805247i$	$\mp 1.70419i$	Stable
$0.2$		$1.32971 \times 10^{ - 9}$	$7.3966\,3 \times 10^{ - 10}$	$- 5.88058 \times 10^{ - 9} \mp 1.70419i$	$2.77652 \times 10^{ - 9} \mp 0.805247i$	Unstable
		$0$	$0$	$\mp 0.762301i$	$\mp 1.72908i$	Stable
$0$	$0.03$	$1.32971 \times 10^{ - 9}$	$7.3966\,3 \times 10^{ - 10}$	$- 5.6177 \times 10^{ - 9} \mp 1.72908i$	$2.51365 \times 10^{ - 9} \mp 0.762301i$	Unstable

**Table 11:** Showing roots of the characteristic equation for ARCHIRD with different values of oblateness and P-R drag q_1_ = 0.9971 and q_2_ = 0.9997, a = 0.1269 and e = 0.5117.

A_1_	A_2_	W_1_	W_2_	Stability of triangular equilibrium points		Remark
				$\lambda_{1,2}$	$\lambda_{3,4}$	
$0$	$0$	$0$	$0$	$\mp 0.467484i$	$\mp 2.33498i$	Stable
		$2.59295\, \times 10^{ - 8}$	$1.75056\, \times 10^{ - 9}$	$- 5.3616\, \times 10^{ - 8} \mp 2.33498i$	$1.20959\, \times 10^{ - 8} \mp 0.467484i$	Unstable
$0.0001$	$0.0003$	$0$	$0$	$\mp 0.467945i$	$\mp 2.3349i$	Stable
		$2.59295\, \times 10^{ - 8}$	$1.75056\, \times 10^{ - 9}$	$- 5.36212 \times 10^{ - 8} \mp 2.3349i$	$1.21012 \times 10^{ - 8} \mp 0.467945i$	Unstable
$0.001$	$0.003$	$0$	$0$	$\mp 0.472079i$	$\mp 2.33419i$	Stable
		$2.59295\, \times 10^{ - 8}$	$1.75056\, \times 10^{ - 9}$	$- 5.36688 \times 10^{ - 8} \mp 2.33419i$	$1.21488 \times 10^{ - 8} \mp 0.472079i$	Unstable
$0.01$	$0.03$	$0$	$0$	$\mp 0.511849i$	$\mp 2.32701i$	Stable
		$2.59295\, \times 10^{ - 8}$	$1.75056\, \times 10^{ - 9}$	$- 5.41552 \times 10^{ - 8} \mp 2.32701i$	$1.26352 \times 10^{ - 8} \mp 0.511849i$	Unstable
$0.01$	$0$	$0$	$0$	$\mp 0.471186i$	$\mp 2.33356i$	Stable
		$2.59295\, \times 10^{ - 8}$	$1.75056\, \times 10^{ - 9}$	$- 5.3667 \times 10^{ - 8} \mp 2.33356i$	$1.21469 \times 10^{ - 8} \mp 0.471186i$	Unstable
$0.1$	$0$	$0$	$0$	$\mp 0.50368i$	$\mp 2.32066i$	Stable
		$2.59295\, \times 10^{ - 8}$	$1.75056\, \times 10^{ - 9}$	$- 5.41404 \times 10^{ - 8} \mp 2.32066i$	$1.26204 \times 10^{ - 8} \mp 0.50368i$	Unstable
$0.2$	$0$	$0$	$0$	$\mp 0.538309i$	$\mp 2.30604i$	Stable
		$2.59295\, \times 10^{ - 8}$	$1.75056\, \times 10^{ - 9}$	$- 5.46986 \times 10^{ - 8} \mp 2.30604i$	$1.31786 \times 10^{ - 8} \mp 0.538309i$	Unstable
		$0$	$0$	$\mp 0.508373i$	$\mp 2.32845i$	Stable
$0$	$0.03$	$2.59295\, \times 10^{ - 8}$	$1.75056\, \times 10^{ - 9}$	$- 5.41016 \times 10^{ - 8} \mp 2.32845i$	$1.25816 \times 10^{ - 8} \mp 0.508373i$	Unstable

**Table 12:** Showing roots of the characteristic equation with different values of radiation and P-R drag with A_1_ = 0.1, A_2_ = 0.2, a = 0.5 and e = 0.5

A_1_	A_2_	W_1_	W_2_	Stability of triangular equilibrium points		Remark
				$\lambda_{1,2}$	$\lambda_{3,4}$	
1	1	$0$	$0$	$\mp 0.828138i$	$\mp 2.24676i$	Stable
1	1	$2.59295\, \times 10^{ - 8}$	$1.7505591\, \times 10^{ - 9}$	$- 6.04802 \times 10^{ - 8} \mp 2.24676i$	$1.89602 \times 10^{ - 8} \mp 0.828138i$	Unstable
1	1	$1.32971\, \times 10^{ - 9}$	$0$	$- 0.299825 \mp 1.37186i$	$0.299825 \mp 1.37186i$	Unstable
1	1	$0$	$7.3966346\, \times 10^{ - 10}$	$- 0.299825 \mp 1.37186i$	$0.299825 \mp 1.37186i$	Unstable
1	0.9997	$0$	$0$	$\mp 0.828153i$	$\mp 2.24675i$	Stable
1	0.9997	$2.59295\, \times 10^{ - 8}$	$1.75056 \times 10^{ - 9}$	$- 6.04845 \times 10^{ - 8} \mp 2.24675i$	$1.89606 \times 10^{ - 8} \mp 0.828153i$	Unstable
1	0.99986	$1.32971\, \times 10^{ - 9}$	$0$	$- 0.299837 \mp 1.37187i$	$0.299837 \mp 1.37187i$	Unstable
1	0.99986	0	$7.39663\, \times 10^{ - 10}$	$- 0.299837 \mp 1.37187i$	$0.299837 \mp 1.37187i$	Unstable
0.9971	1	$0$	$0$	$\mp 0.828282i$	$\mp 2.2467i$	Stable
0.9971	1	$1.32971\, \times 10^{ - 9}$	$1.75056 \times 10^{ - 9}$	$- 6.04845 \times 10^{ - 8} \mp 2.2467i$	$1.89645 \times 10^{ - 9} \mp 0.828282i$	Unstable
0.9997	1	$0$	$7.39663\, \times 10^{ - 10}$	$- 0.299844 \mp 1.37187i$	$0.299844 \mp 1.37187i$	Unstable
0.9997	1	$1.32971\, \times 10^{ - 9}$	$7.39663\, \times 10^{ - 10}$	$- 0.299844 \mp 1.37187i$	$0.299844 \mp 1.37187i$	Unstable
0.9971	0.9997	$0$	$0$	$\mp 0.828297i$	$\mp 2.2467i$	Stable
0.9971	0.9997	$2.59295\, \times 10^{ - 8}$	$1.75056 \times 10^{ - 9}$	$- 6.0485 \times 10^{ - 8} \mp 2.2467i$	$1.8965 \times 10^{ - 8} \mp 0.828297i$	Unstable
0.9997	0.99986	$1.32971\, \times 10^{ - 9}$	$0$	$- 0.299855 \mp 1.37187i$	$0.299855 \mp 1.37187i$	Unstable
0.9997	0.99986	$0$	$7.3966346\, \times 10^{ - 10}$	$- 0.299855 \mp 1.37187i$	$0.299855 \mp 1.37187i$	Unstable

## Conclusion

We have studied the positions and stability of triangular equilibrium points in the elliptic restricted three body problem (ER3BP) under the speculation that the primary bodies are both oblate and luminous with Poynting Robertson (P-R) drag. It is seen that the equations of motion for the stated problem are affected by the oblateness, semi-major axis, radiation pressure, eccentricity of the orbits, and Poynting Robertson (P-R) drag.

The stability of the system has been investigated using the analytic and numerical approach.

The locations and stability of triangular points are significantly affected by the aforesaid Parameters. The system is stable in the presence of oblateness or radiation or both of any of the primaries or both primaries. But it becomes unstable when the P-R drag of any or both of the primary bodies come into action, whether one or both of the primaries are oblate or luminous or both. Thus, Poynting Robertson (P-R) drag ruins the stability.

## Data Availability

All data generated or analyzed during the current study are included in this manuscript.
